# Supplementary material for: Network-based machine learning approach to predict immunotherapy response in cancer patients
Source: Nat Commun. 2022 Jun 28;13:3703. doi: 10.1038/s41467-022-31535-6 (PMC9240063; doi:10.1038/s41467-022-31535-6)
Supplement: Supplementary file 1 — Supplementary Information [file 41467_2022_31535_MOESM1_ESM.pdf]

# Supplementary Information

## Title: Network-based machine learning approach to predict immunotherapy response in cancer patients

To whom correspondence should be addressed:

Sanguk Kim, Ph.D.

Department of Life Sciences, Pohang University of Science and Technology, Pohang 790-784, Korea; E-mail: sukim@postech.ac.kr; Tel: +82-54-279-2348; Fax: +82-54-279-2199

## Table of contents

Supplementary Information files contain 35 Supplementary Figures and 6 Supplementary Table

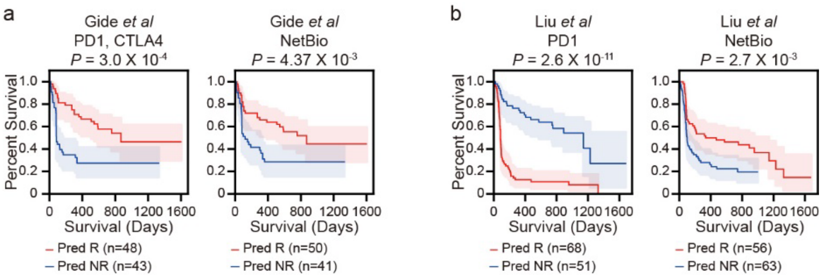


**Supplementary Figure 1. Prediction of progression free survival (PFS) in (a) Gide and (b) Liu dataset.** The log-rank test was used to measure statistical significance. The light-colored areas indicated 95% confidence interval of each percent survival.

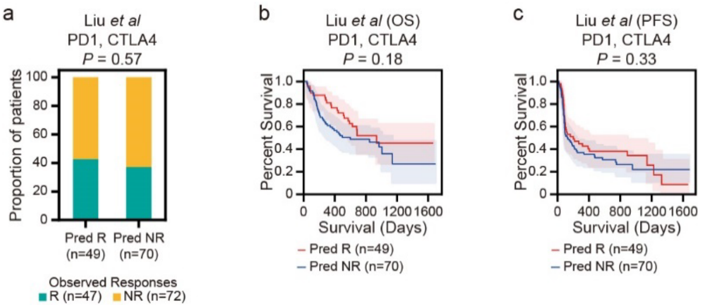


**Supplementary Figure 2. Prediction of drug response, overall survival (OS), and progression free survival (PFS) in the Liu dataset using expression levels of PD1 and CTLA4.** (**a**) Immunotherapy response prediction using the expression levels of PD1 and CTLA4. The proportions of observed responders (teal) and non-responders (orange) among predicted responders (Pred R) and non-responders (Pred NR) are shown. The two-sided Fisher's exact test was used to compute statistical significance. (**b**)–(**c**) The (b) OS and (c) PFS of predicted responders and non-responders based on LOOCV. The predicted responders and non-responders are depicted in red and blue, respectively. The log-rank test was used to measure statistical significance. The light-colored areas indicated 95% confidence interval of each percent survival.

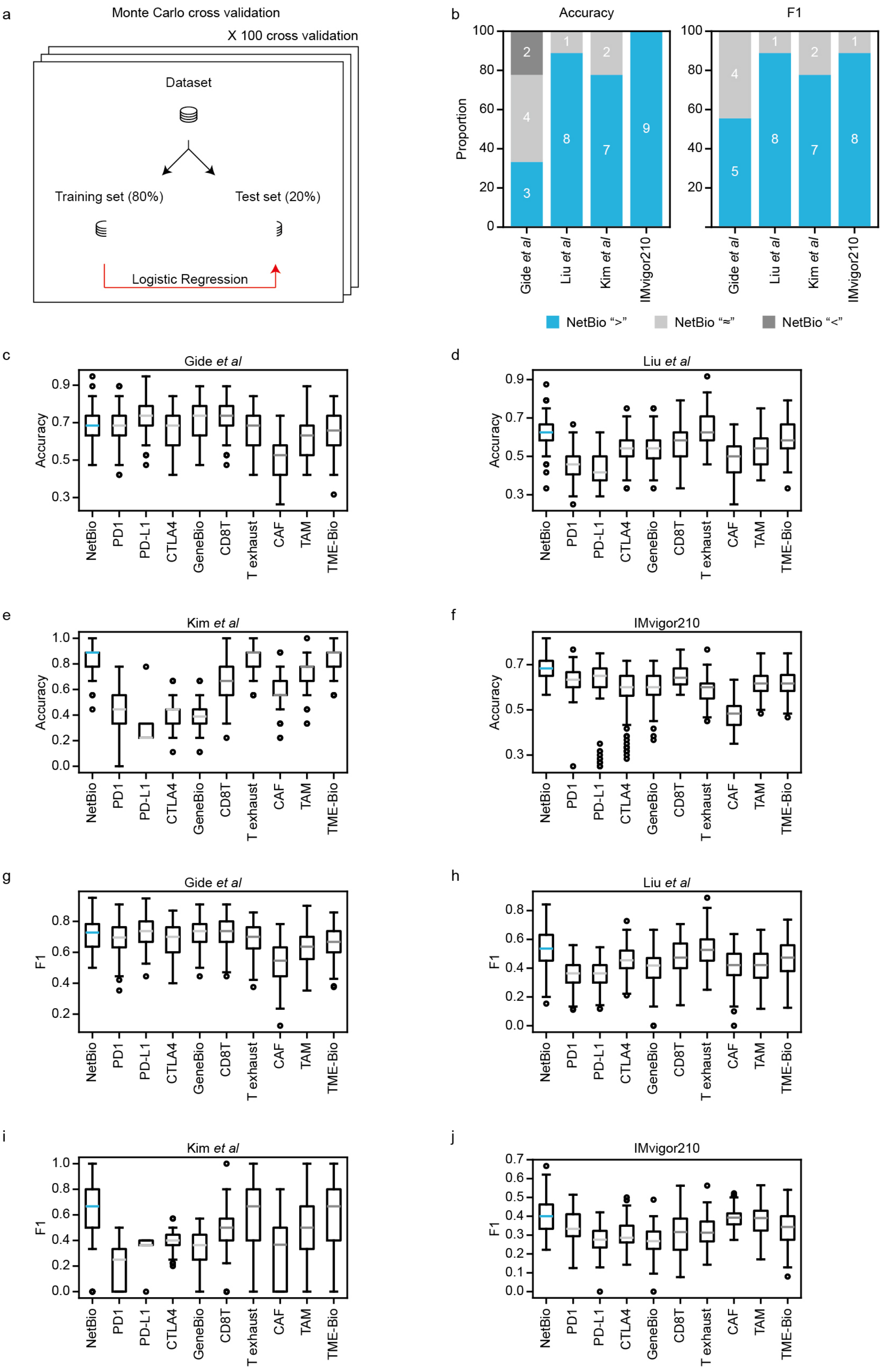


**Supplementary Figure 3. Prediction performances using fewer training samples,**

**computed using Monte Carlo cross-validation.** (**a**) Overall scheme of Monte Carlo cross-validation. (**b**) Summarized classification results (two-sided Student's t-test $P < 0.05$ considered significant) for 56 independent tests. (**c**)–(**j**) Boxplots of prediction performances in four different cohorts, using accuracy or the F1 score as a metric to quantify prediction performance. 91, 119, 45, 298 independent samples were used for Gide, Liu, Kim and IMvigor210 datasets, respectively. Boxplot shows median value, interquartile range (IQR) as bounds of the box and whiskers that extends from the box to upper/lower quartile $\pm$ IQR$\times$1.5.

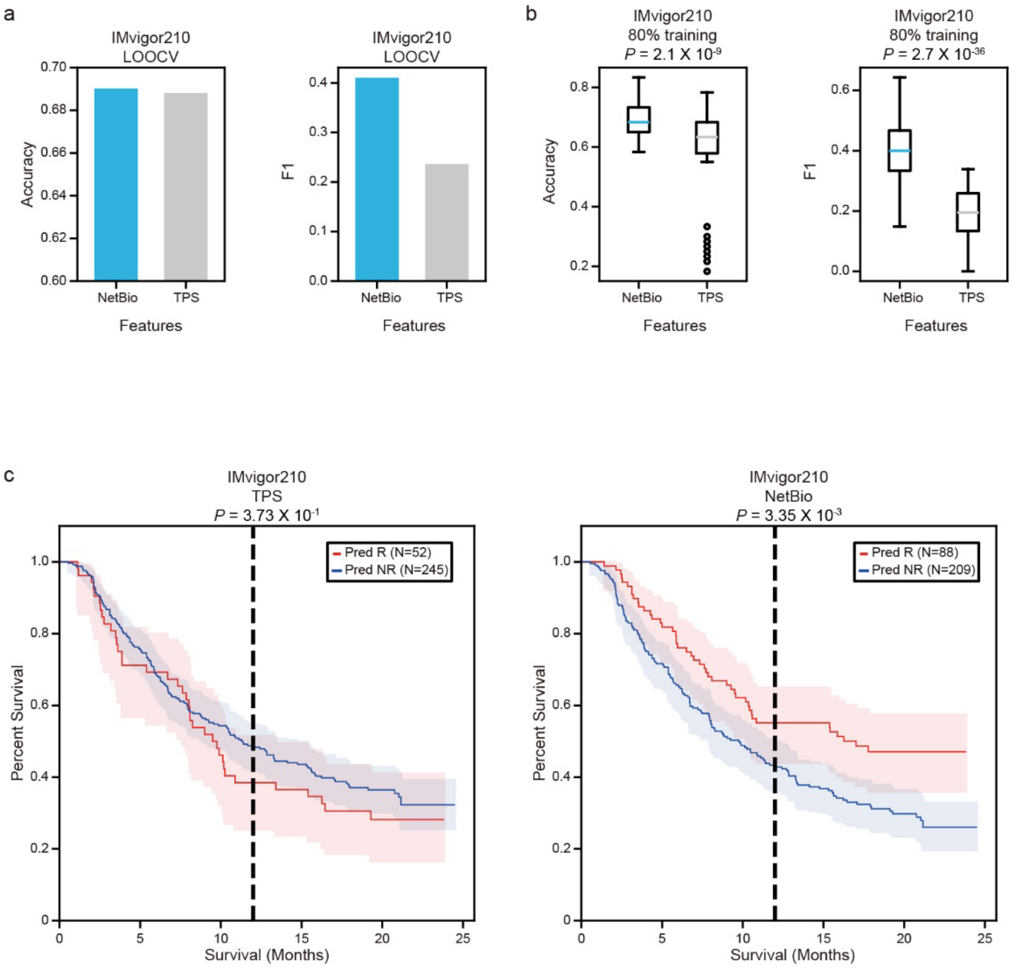


**Supplementary Figure 4. Comparing NetBio-based and tumor proportion score (TPS)-based predictions**. (**a**) Predictive performance of Leave-One-Out Cross-Validation (LOOCV). (**b**) Predictive performance of Monte-Carlo cross-validation. 298 independent samples were used. Eighty percent of the samples were used as the training set, and the remaining 20% were used as the test set for 100 independent iterations. Statistical significance was measured using the two-sided Student's t-test. Boxplot shows median value, interquartile range (IQR) as bounds of the box and whiskers that extends from the box to upper/lower quartile $\pm$ IQR$\times$1.5. (**c**) Prediction of overall survival using either TPS or NetBio to train a machine-learning model (Logistic Regression). Statistical significance was measured using log-rank test. The light-colored areas indicated 95% confidence interval of each percent survival. Pred R, Predicted

Responders. Pred NR, Predicted Non-Responders.

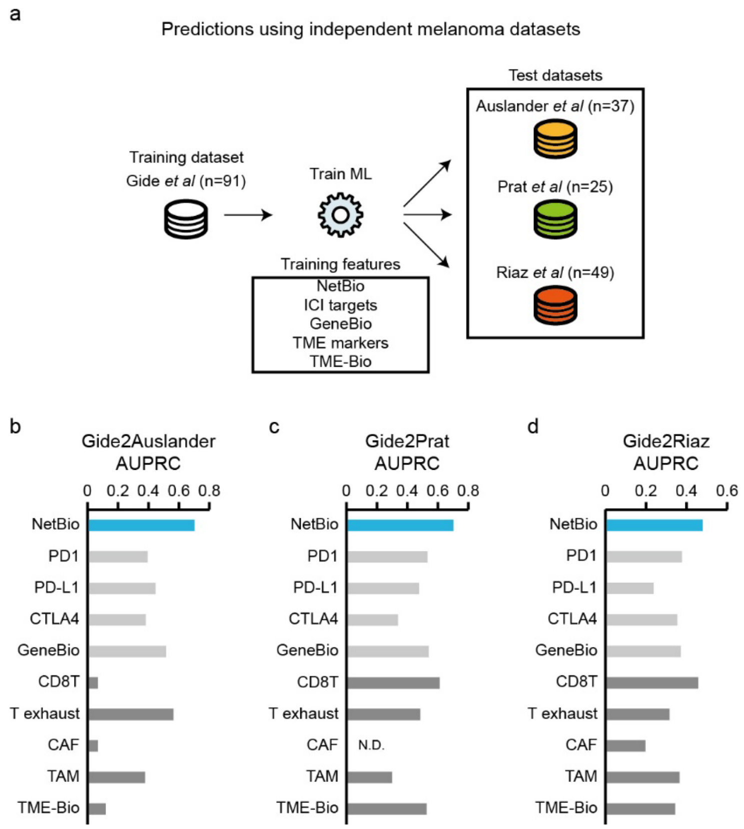


**Supplementary Figure 5. Predictive performance in three independent melanoma datasets**. (**a**) Overall scheme of immunotherapy response prediction in three independent datasets. The datasets used to train and test the machine-learning models, transcriptomic features used to train the models, and number of samples for each dataset are displayed. (**b**)–(**d**) The area under the precision-recall curve (AUPRC) for the (b) Auslander, (c) Prat, and (d) Riaz datasets are shown. No expression profiles of cancer-associated fibroblast (CAF) marker genes were available in the Prat dataset. N.D., Not detected.

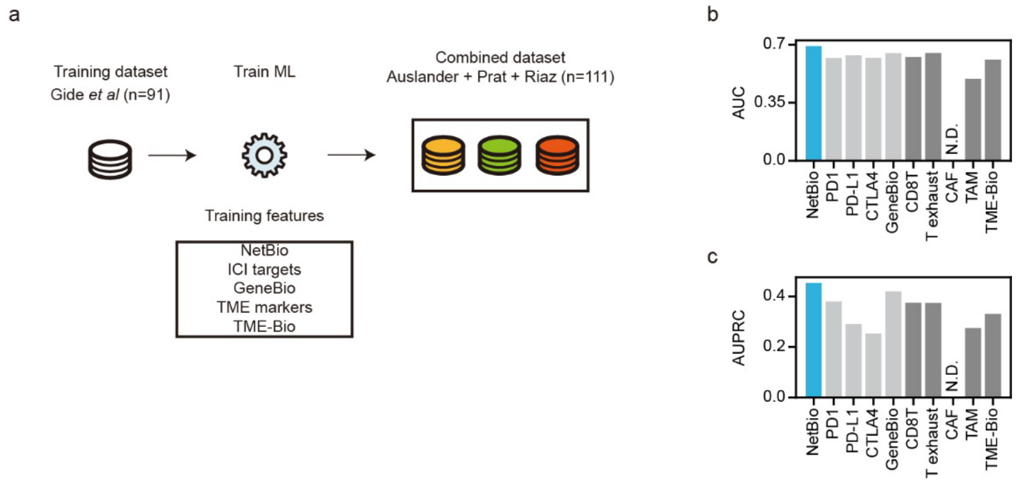


**Supplementary Figure 6. Predictive performance in combined melanoma datasets**. (**a**) Overall scheme of immunotherapy response prediction. (**b**)–(**c**) Predictive performance using (a) area under the curve (AUC) or (b) area under the precision-recall curve AUPRC. No expression profiles of cancer-associated fibroblast (CAF) marker genes were available in the Prat dataset. N.D., Not Determined.

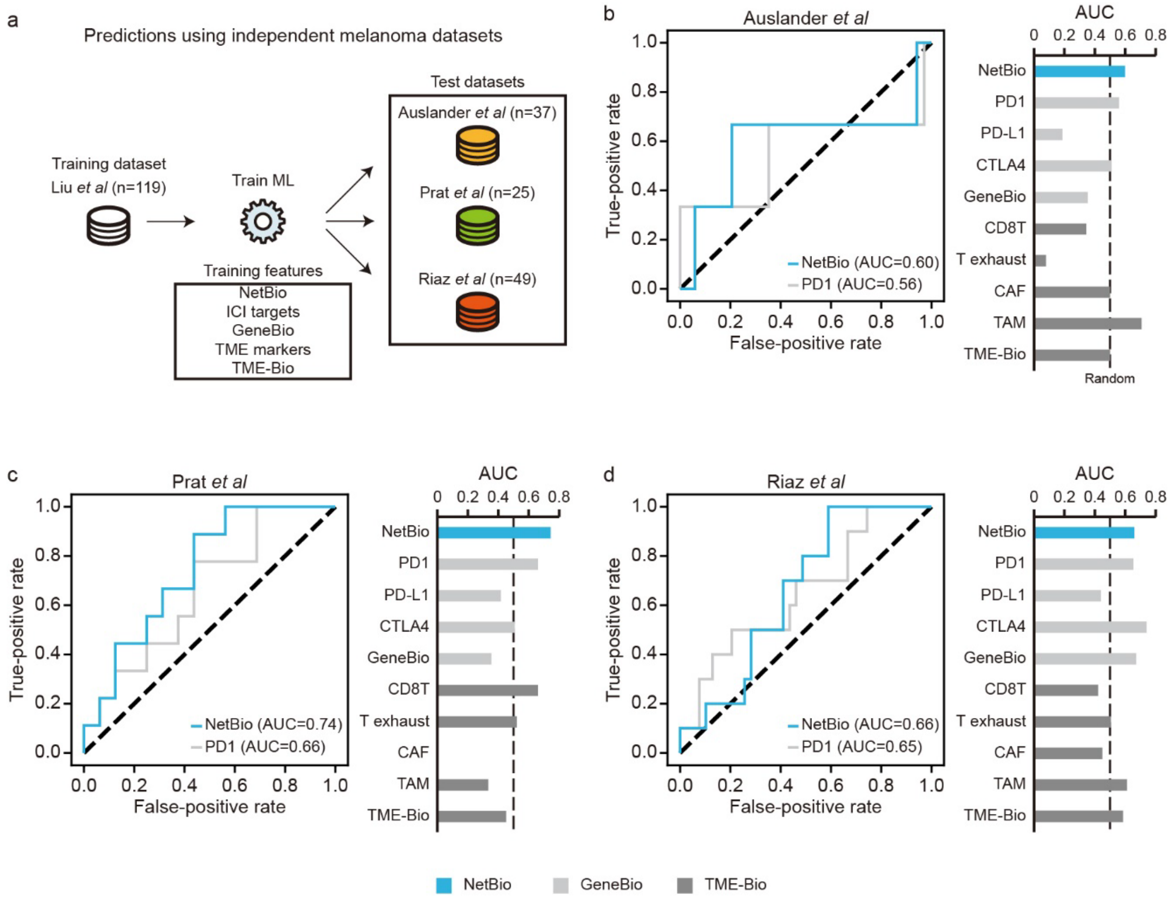


**Supplementary Figure 7. Predictive performance in three independent melanoma datasets.** (**a**) Overall scheme of immunotherapy response prediction in three independent datasets. The Liu dataset was used to train a machine-learning models. (**b**)–(**d**) The area under the receiver operating characteristic curve (AUC) for the (b) Auslander, (c) Prat, and (d) Riaz datasets is shown. The random expectation, equaling an AUC of 0.5, is displayed as dotted lines. No expression profiles of cancer-associated fibroblast (CAF) marker genes were available in the Prat dataset. N.D., Not detected.

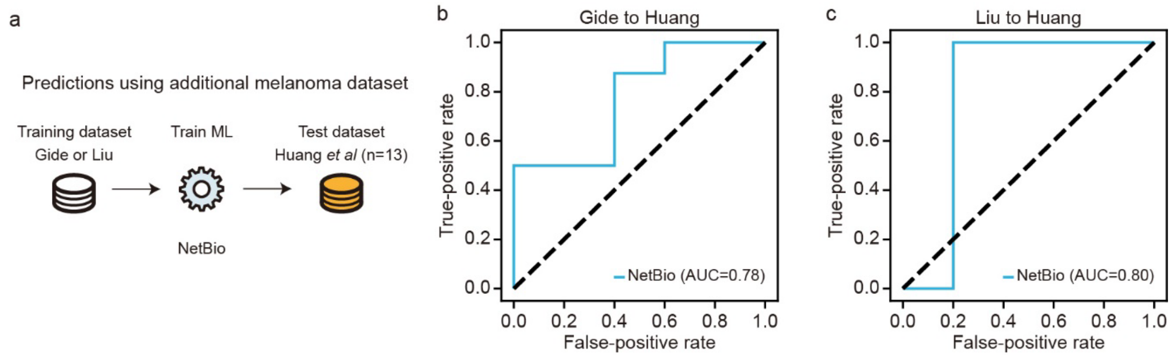


**Supplementary Figure 8. Performance of across-study predictions in an additional cohort.** (**a**) Overall scheme of immunotherapy response prediction in an independent dataset. (**b**)–(**c**) The area under the receiver operating characteristic curve (AUC) for (b) Gide to Huang and (c) Liu to Huang. The random expectation is displayed as dotted lines.

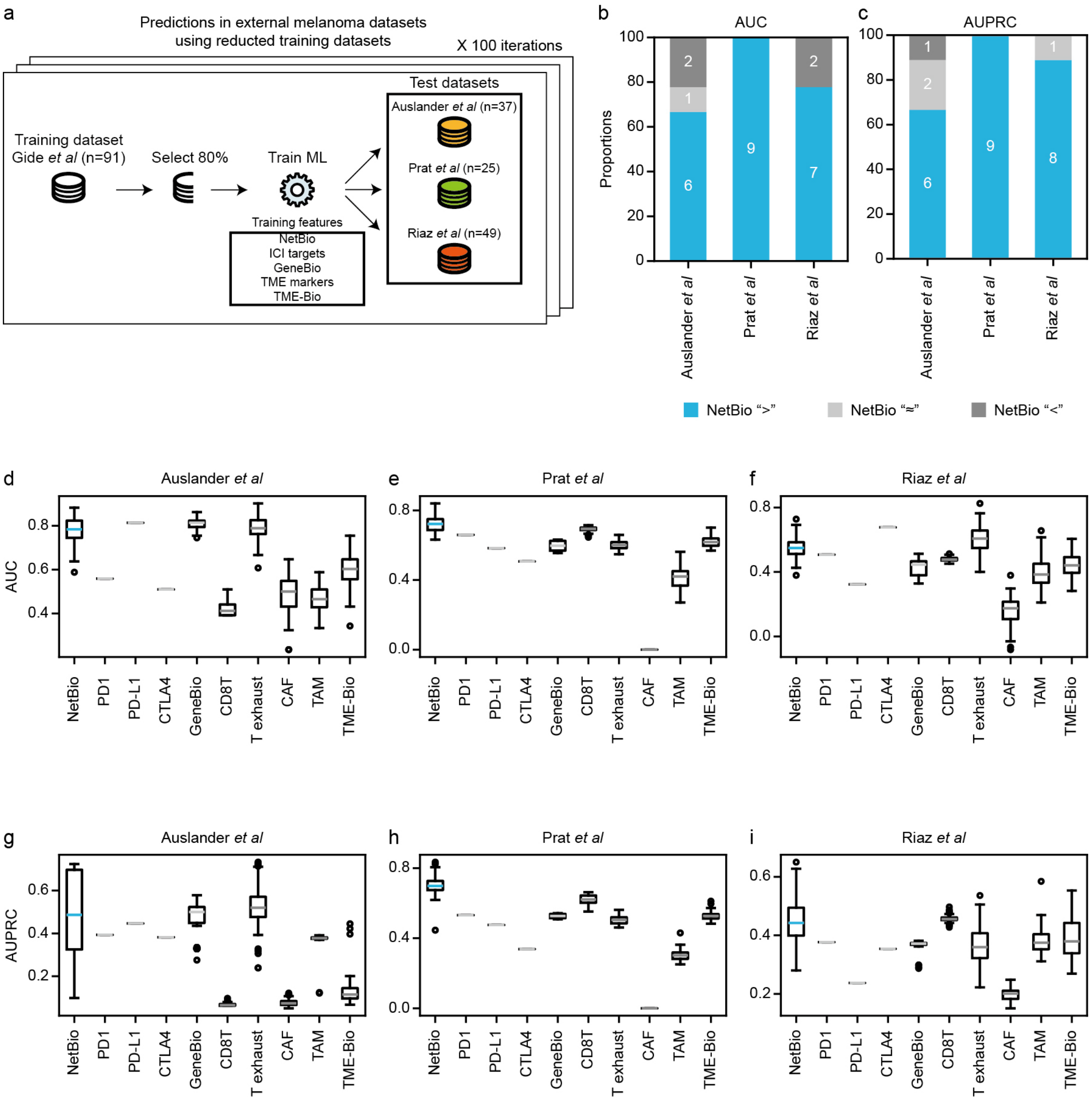


**Supplementary Figure 9. Prediction performances using fewer training samples to predict immunotherapy responses in external melanoma datasets.** (**a**) Overall scheme for predictions in external melanoma datasets. (**b**)–(**c**) Summarized classification results (two-sided Student's t-test; $P < 0.05$ was considered significant) using (b) AUC and (c) AUPRC as a metric to quantify prediction performance. (**d**)–(**i**) Boxplots of prediction performances in three different cohorts, using (d)–(f) AUC and (g)–(i) AUPRC as a metric to quantify prediction performance. 37, 25 and 49 independent samples were used for Auslander, Prat and Riaz

datasets, respectively. Boxplot shows median value, interquartile range (IQR) as bounds of the box and whiskers that extends from the box to upper/lower quartile $\pm$ IQR$\times$1.5.

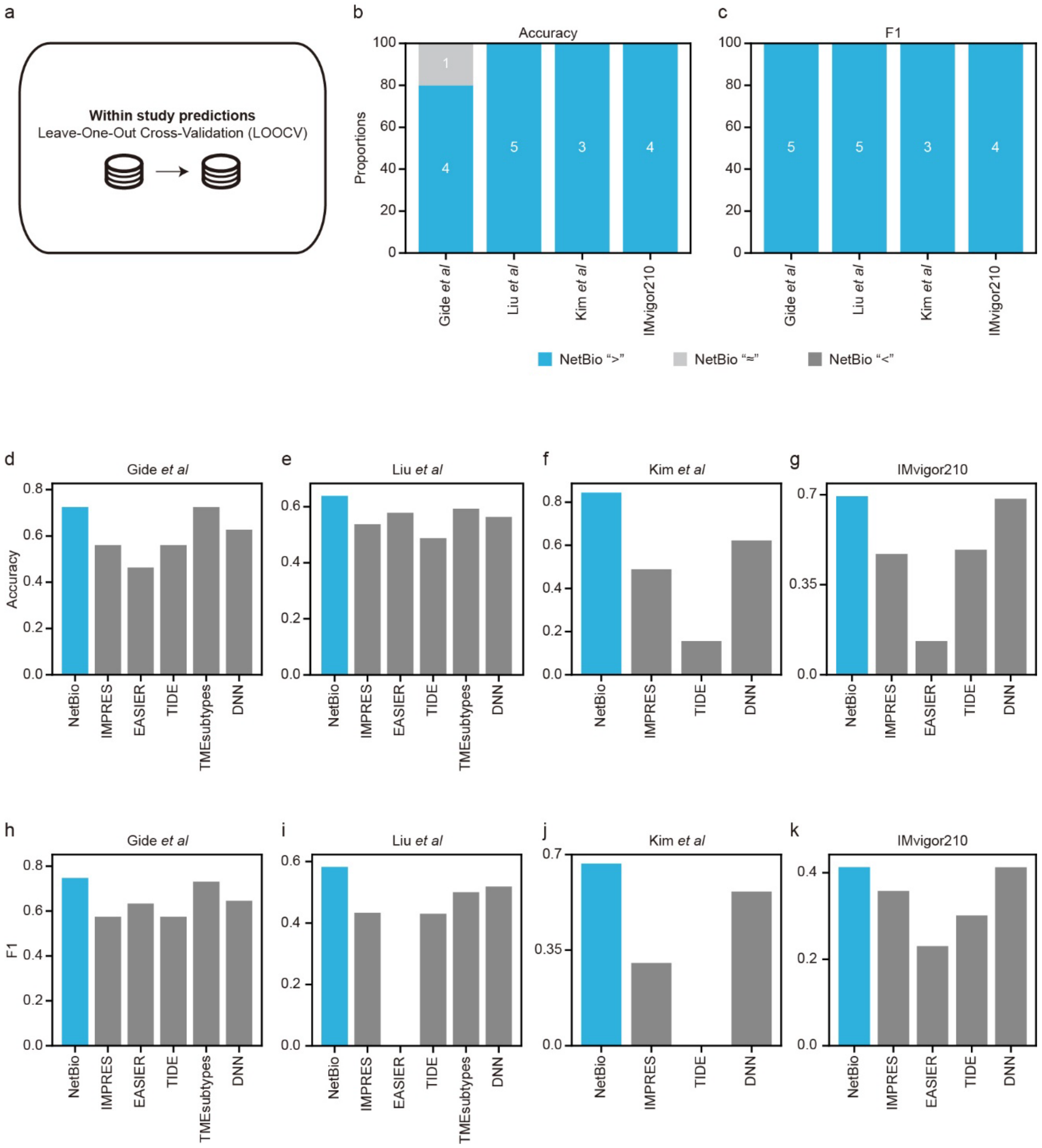


**Supplementary Figure 10. Comparison of Leave-One-Out Cross-Validation (LOOCV) performances**. (**a**) Overall scheme of immunotherapy response prediction using LOOCV. (**b**)–(**c**) Summarized classification results using (b) accuracy and (c) F1 score as a metric to quantify predictive performance. (**d**)–(**k**) LOOCV predictive performances based on (d)–(g) accuracy and (h)–(k) F1 score.

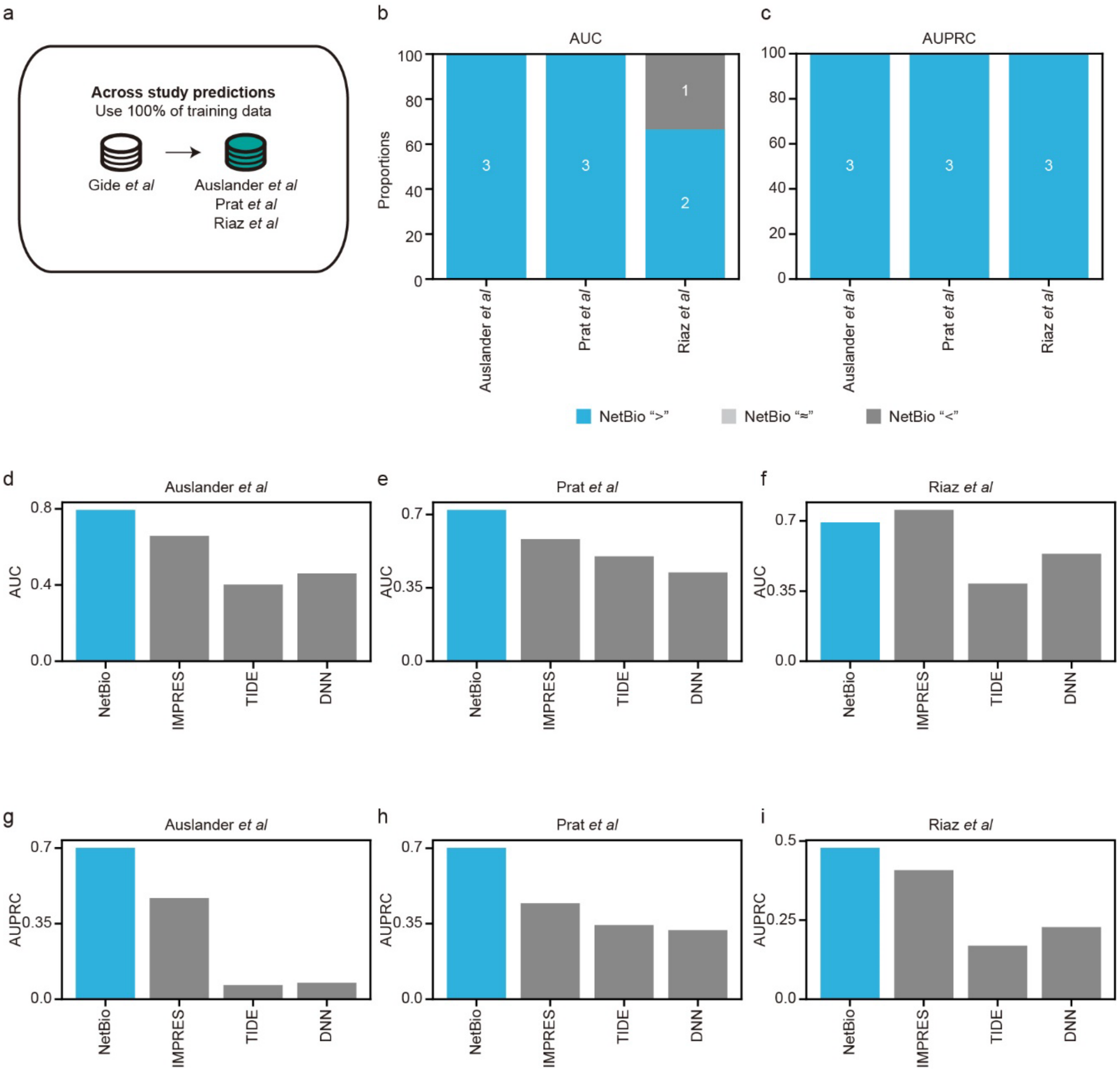


**Supplementary Figure 11. Comparison of across-study predictive performance**. (**a**) Overall scheme of across-study predictions of immunotherapy response. (**b**)–(**c**) Summarized prediction results using (b) area under the receiver operating characteristic curve (AUC) and (c) area under the precision-recall curve (AUPRC) as a metric to quantify predictive performance. (**d**)–(**k**) Across-study predictive performance based on (d)–(g) AUC and (h)–(k) AUPRC.

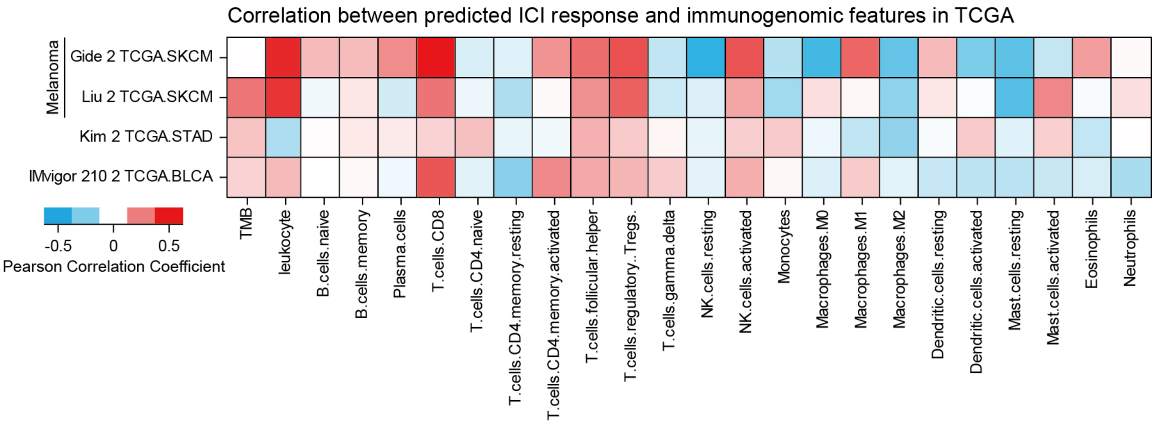


**Supplementary Figure 12. Correlation between NetBio-based predictions and immunogenic features in the TCGA cohort.** Correlation was measured using Pearson's correlation coefficient (PCC).

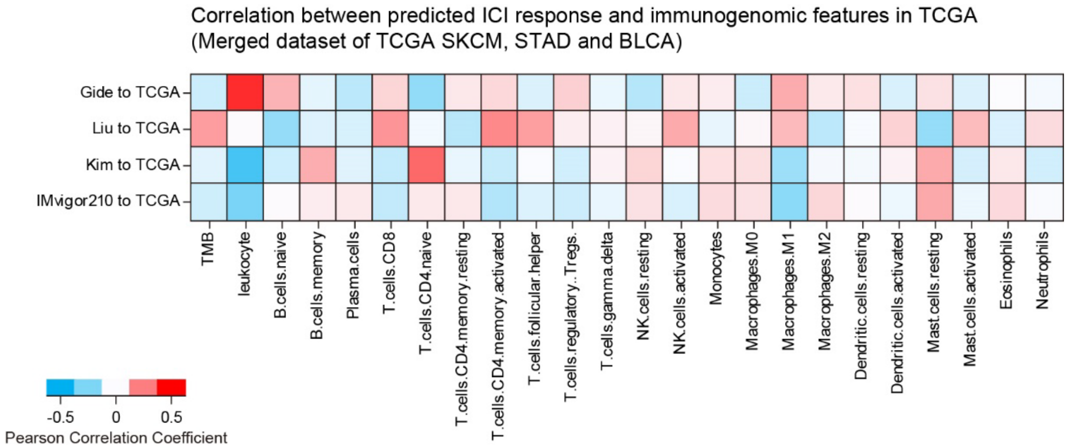


**Supplementary Figure 13. Correlation between NetBio-based predictions and immunogenic features in a merged TCGA cohort comprising melanoma (SKCM), gastric cancer (STAD), and bladder cancer (BLCA).** Correlation was measured using Pearson's correlation coefficient.

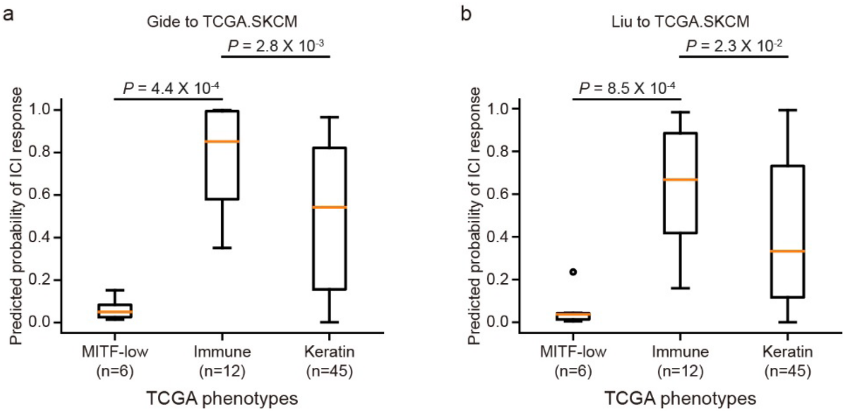


**Supplementary Figure 14. Association between predicted ICI response and TCGA subtypes in melanoma patients.** (**a**)–(**b**) Associations measured using the (a) Gide or (b) Liu cohort to train a NetBio-based machine-learning model. Statistical significance was measured using the two-sided Mann-Whitney U test. Boxplot shows median value, interquartile range (IQR) as bounds of the box and whiskers that extends from the box to upper/lower quartile ± IQR×1.5.

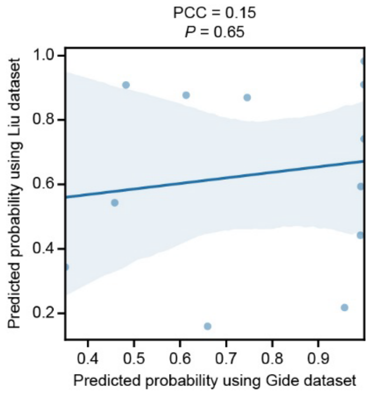


**Supplementary Figure 15. Correlation between predicted ICI responses of TCGA melanoma patients with the immune subtype based on different training datasets.** Associations between ICI responses predicted using the Gide or Liu datasets to train a NetBio-based machine-learning model. Pearson correlation was used to statistical significance. The light-colored area indicated 95% confidence interval of linear regression line.

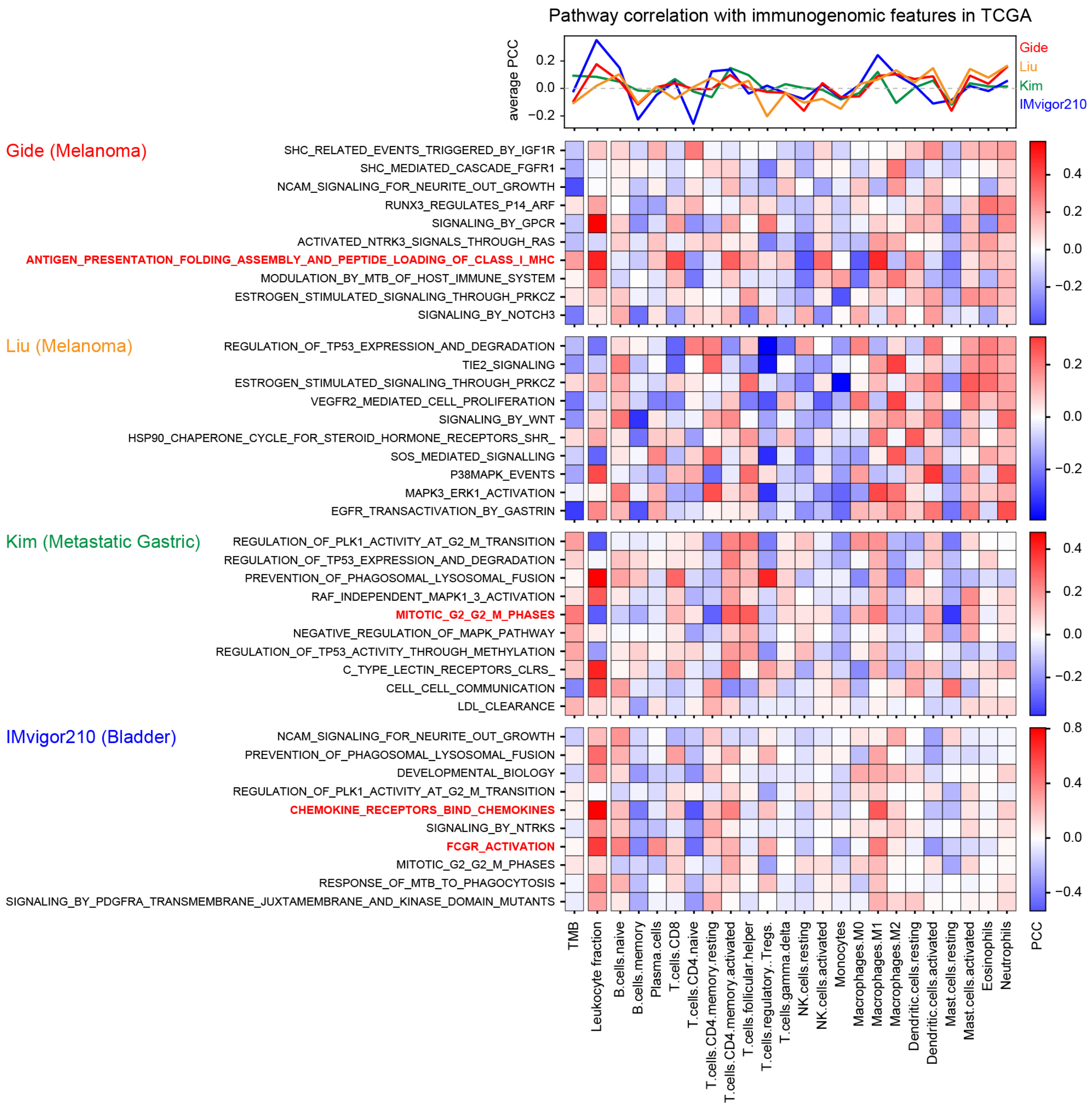


**Supplementary Figure 16. Immunogenomic characteristics of the top 10 pathways with the greatest positive feature importance.** Correlation between the expression level of the top 10 predictive features with positive coefficients and immunogenic signatures in the TCGA dataset are shown. Correlation was measured using Pearson's correlation coefficient (PCC).

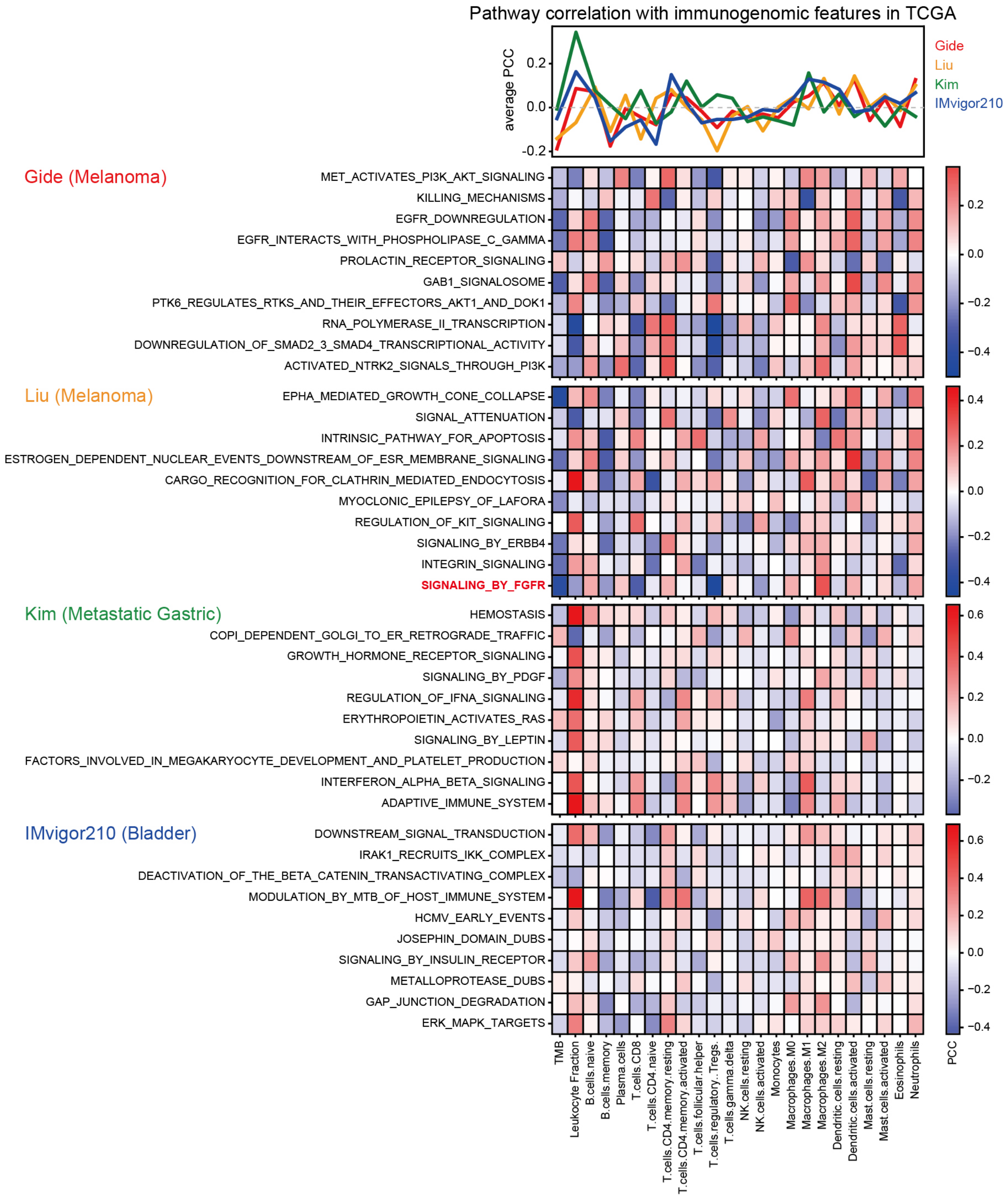


**Supplementary Figure 17. Immunogenomic characteristics of the top 10 pathways with the greatest negative feature importance.** Correlation between the expression level of the top 10 predictive features with negative coefficients and immunogenic signatures in the TCGA dataset are shown. Correlation was measured using Pearson's correlation coefficient (PCC).

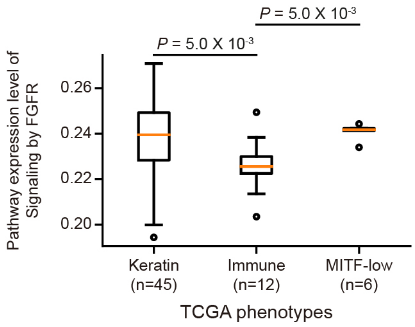


**Supplementary Figure 18. Expression level of 'signaling by FGFR' pathway among TCGA melanoma subtypes.** Statistical significance was measured using the two-sided Mann-Whitney U test. Boxplot shows median value, interquartile range (IQR) as bounds of the box and whiskers that extends from the box to upper/lower quartile $\pm$ IQR×1.5.

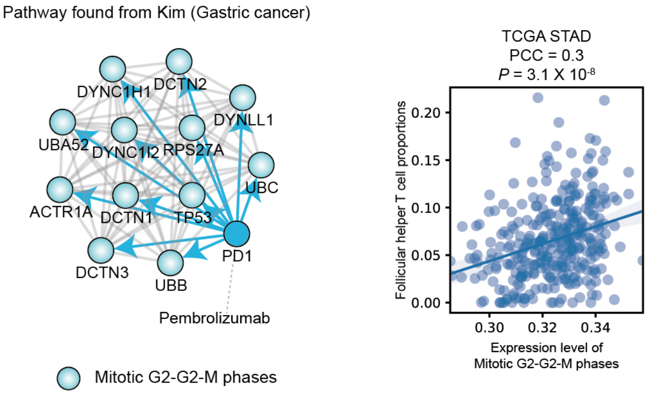


**Supplementary Figure 19. Expression level of a NetBio pathway (Mitotic G2-G2-M phases) is positively correlated with follicular helper T cell proportions in TCGA gastric cancer.** (Left) Network representation of Mitotic G2-G2-M phases and the pembrolizumab target (PD-1). (Right) Correlation between the expression level of Mitotic G2-G2-M phases and proportions of follicular helper T cell in TCGA gastric cancer patients. Correlation coefficient (PCC) and statistical significance were measured using the two-sided Pearson's correlation. The light-gray area indicated 95% confidence interval of linear regression line.

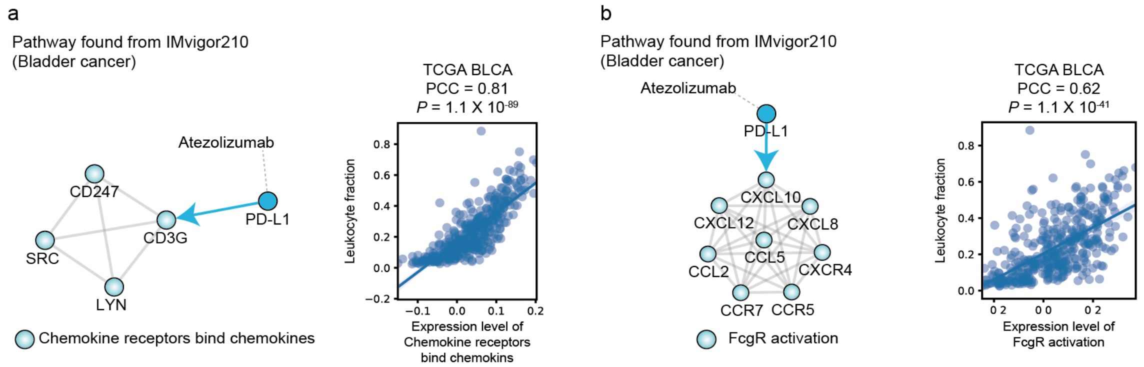


**Supplementary Figure 20. Expression levels of NetBio pathways ("chemokine receptors bind chemokines" and "FcgR activation") are positively correlated with the leukocyte fractions in TCGA bladder cancer.** (**a**)–(**b**) NetBio pathways identified from the IMvigor210 cohort (PD-L1 inhibitor-treated bladder cancer cohort) are shown. Correlation coefficient (PCC) and statistical significance were measured using Pearson's correlation. The light-gray area indicated 95% confidence interval of linear regression line.

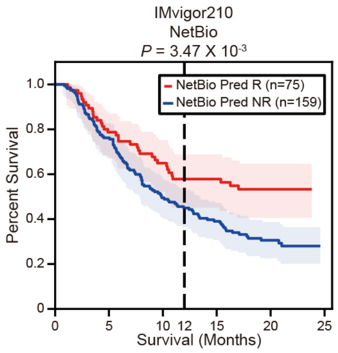


**Supplementary Figure 21. Prediction of overall survival among patients with available TMB levels in the IMvigor210 dataset**. Predictive performance of Leave-One-Out Cross-Validation (LOOCV). Statistical significance was measured using log-rank test. The light-colored areas indicated 95% confidence interval of each percent survival. Pred R, Predicted Responders. Pred NR, Predicted Non-Responders.

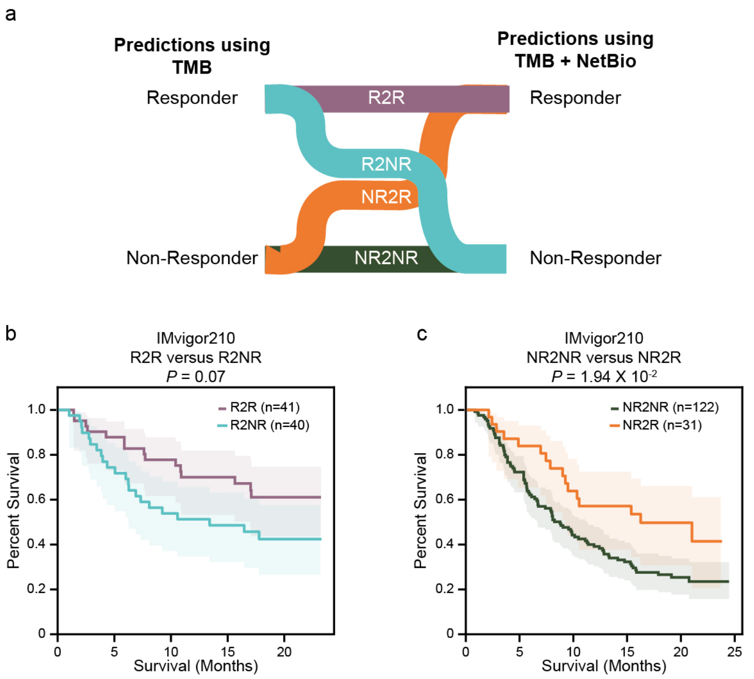


**Supplementary Figure 22. Comparison of predictions from TMB-based PD-L1-inhibitor response prediction and TMB plus NetBio-based prediction.** (**a**) Drug response class changes between TMB-based predictions and TMB- and NetBio-based predictions. (**b**)–(**c**) Overall survival differences between TMB-based predictions and TMB- and NetBio-based predictions. Statistical significances were measured using the log-rank test. The light-colored area indicated 95% confidence interval of percent survival.

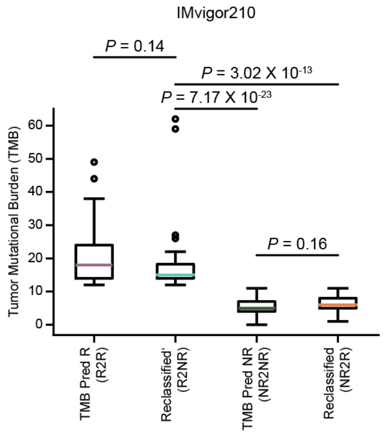


**Supplementary Figure 23. Distribution of TMB levels among predicted ICI responders and non-responders in the IMvigor210 dataset.** The two-sided Mann-Whitney *U* test was used to measure statistical significance. Boxplot shows median value, interquartile range (IQR) as bounds of the box and whiskers that extends from the box to upper/lower quartile ± IQR×1.5. 41, 40, 122 and 31 independent samples were used for R2R, R2NR, NR2NR and NR2R patients, respectively.

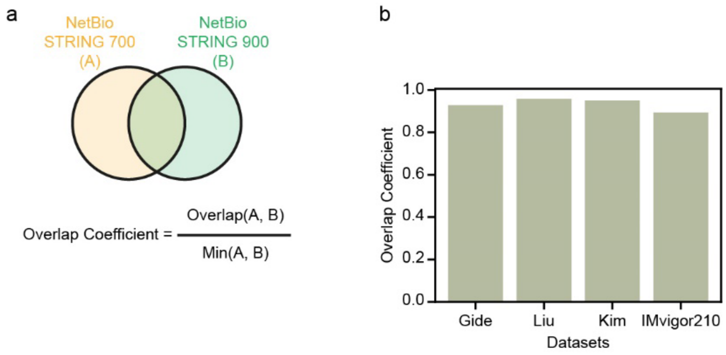


**Supplementary Figure 24. Testing for conservation of NetBio pathways between STRING > 700 and STRING > 900.** (**a**) Overview of similarity calculation using overlap coefficients. (**b**) Similarity of STRING > 700 NetBio pathways and STRING > 900 NetBio pathways.

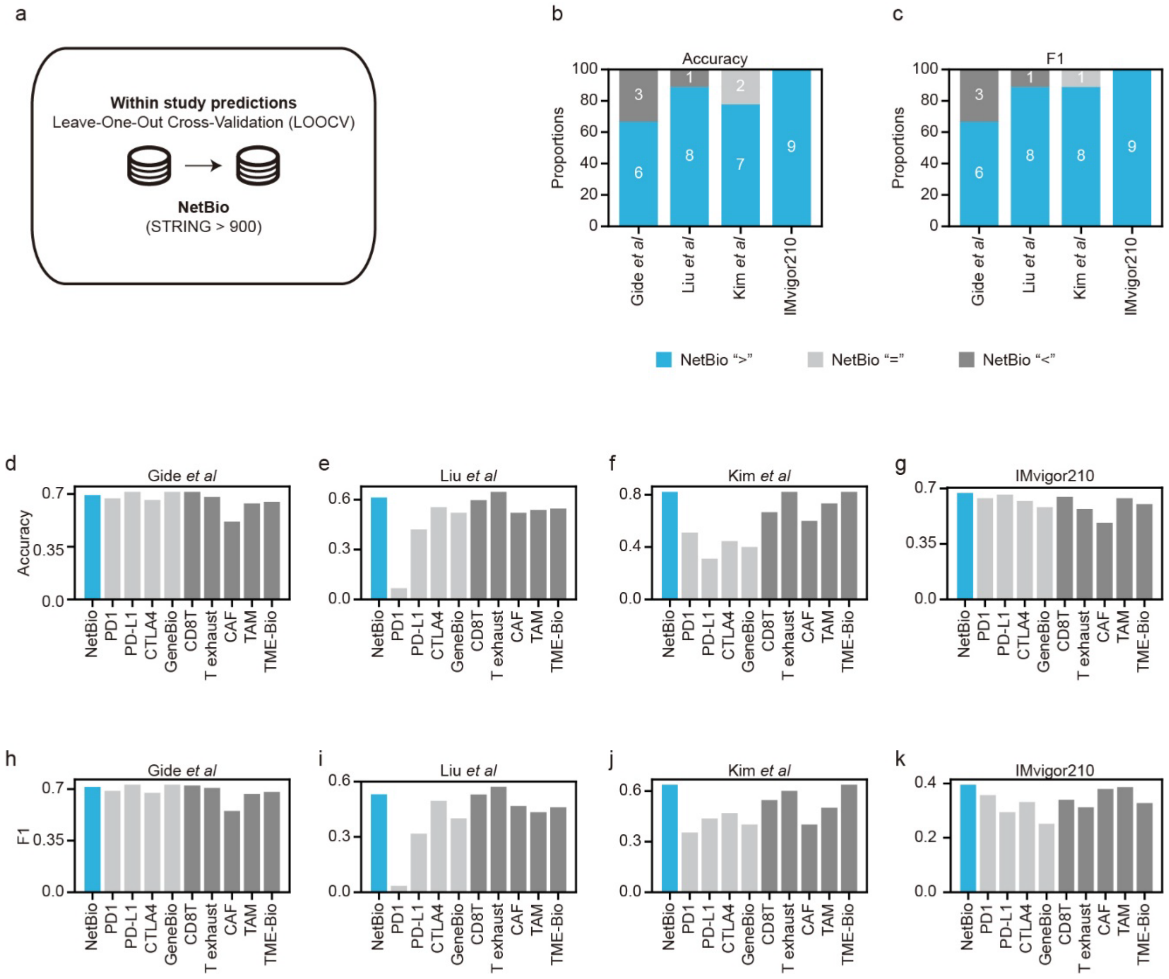


**Supplementary Figure 25. LOOCV predictive performance using the STRING > 900 PPI network.** (**a**) Overall scheme of immunotherapy response prediction using LOOCV. (**b**)–(**c**) Summarized classification results using (b) accuracy or (c) F1 score. (**d**)–(**k**) LOOCV predictive performance using (d)–(g) accuracy and (h)–(k) F1 score.

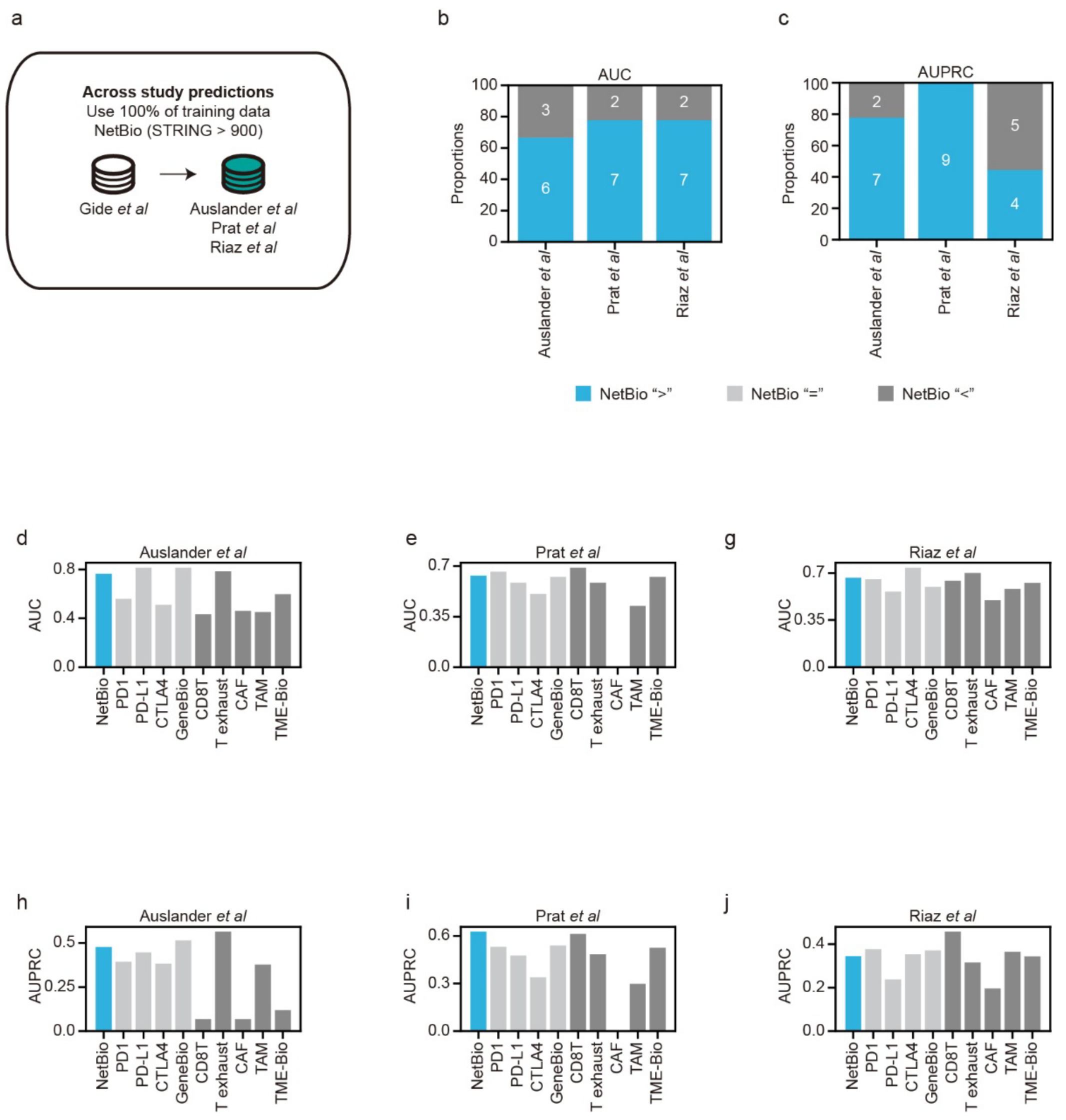


**Supplementary Figure 26. Across-study prediction using the STRING > 900 PPI network.** (**a**) Overall scheme of across-study immunotherapy response prediction. (**b**)–(**c**) Summarized classification results using (b) area under the receiver operating characteristic curve (AUC) or (c) area under the precision-recall curve (AUPRC). (**d**)–(**j**) Across-study predictive performances using (d)-(g) AUC and (h)-(j) AUPRC.

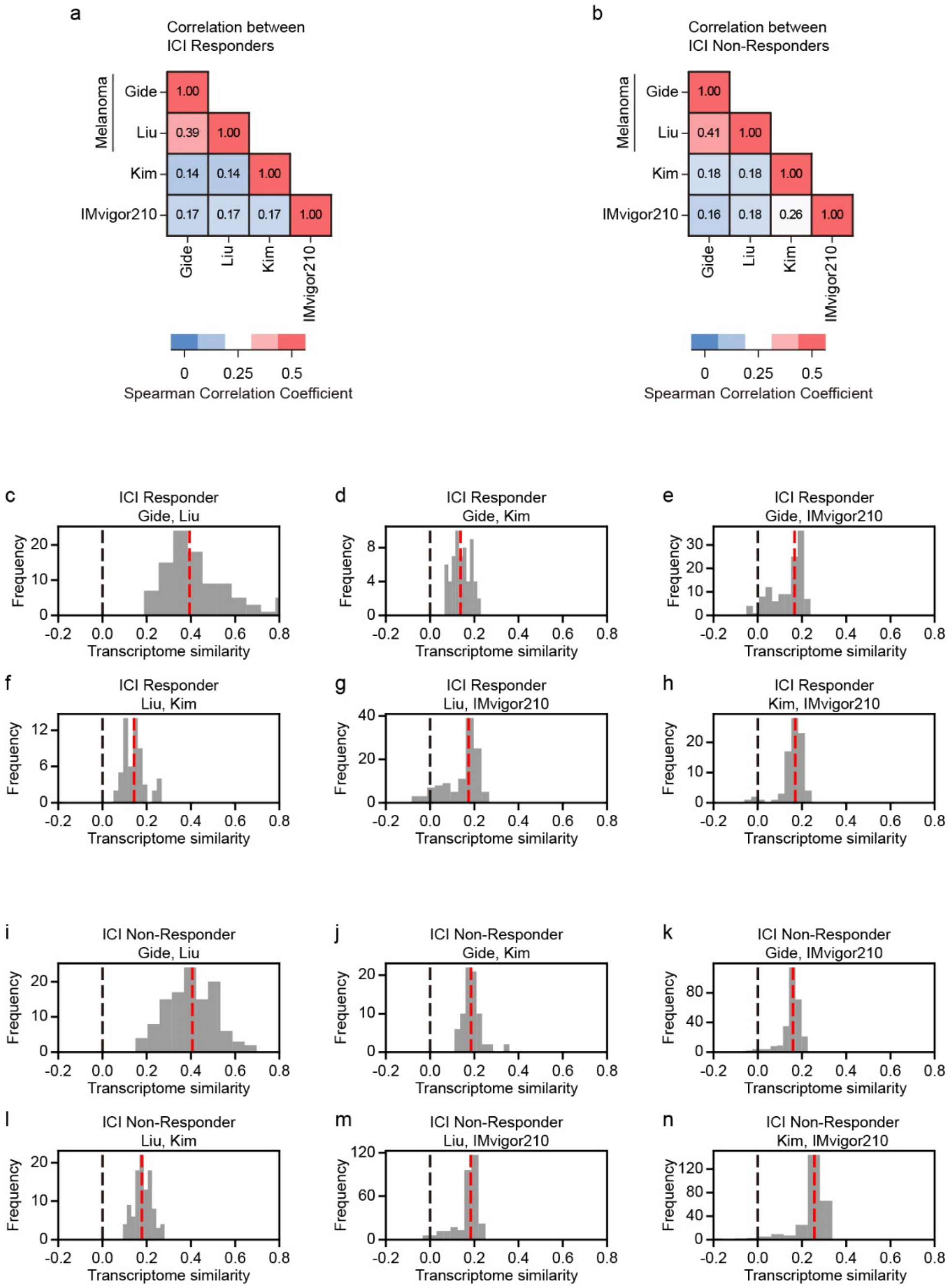


**Supplementary Figure 27. Gene expression similarity between different cohorts. (a)–(b)** Transcriptome similarity among the Gide (melanoma), Liu (melanoma), Kim (metastatic gastric cancer), and IMvigor210 (bladder cancer) cohorts. Spearman correlation was used to measure transcriptome similarity. Median transcriptome similarity from pairwise correlation is shown, where (a) ICI responders and (b) ICI non-responders were used to compute the

similarity. (**c**)–(**n**) Distribution of computed transcriptome similarity between two cohorts. Transcriptome similarity was measured using (c)–(h) ICI responders and (i)–(n) ICI non-responders. Black and red dotted lines denote transcriptome similarity at 0 and at the median of the distribution, respectively.

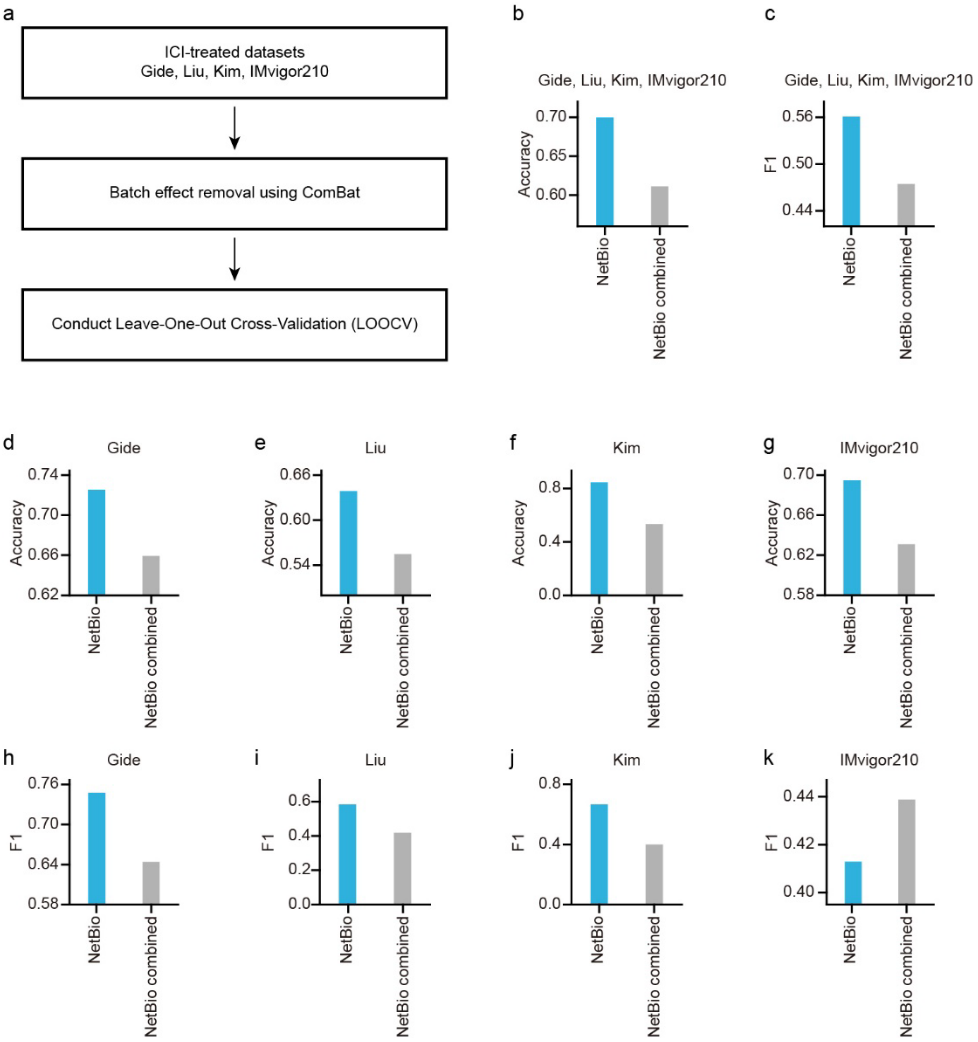


**Supplementary Figure 28. Leave-One-Out Cross-Validation (LOOCV) predictive performance using a combined dataset of all cancer types.** (**a**) Overall scheme for removing batch effects and LOOCV analysis. (**b**)–(**c**) Comparison of the overall predictive performance of NetBio markers and NetBio combined markers using (b) accuracy or (c) F1 score. (**d**)–(**k**) Predictive performance in each cohort using (d)–(g) accuracy or (h)–(k) F1 score.

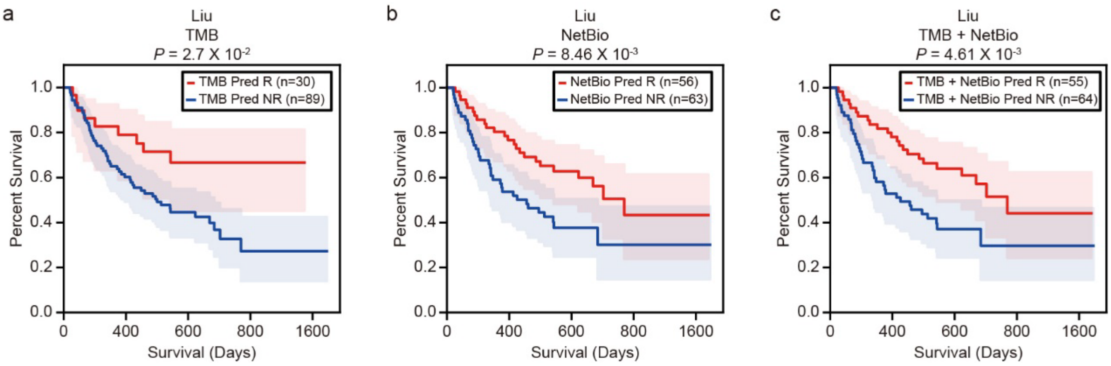


**Supplementary Figure 29. Prediction of overall survival in the Liu dataset.** Prediction using (**a**) TMB, (**b**) NetBio, or (**c**) a combined model (TMB + NetBio). Statistical significance was measured using log-rank test. The light-colored areas indicated 95% confidence interval of each percent survival.

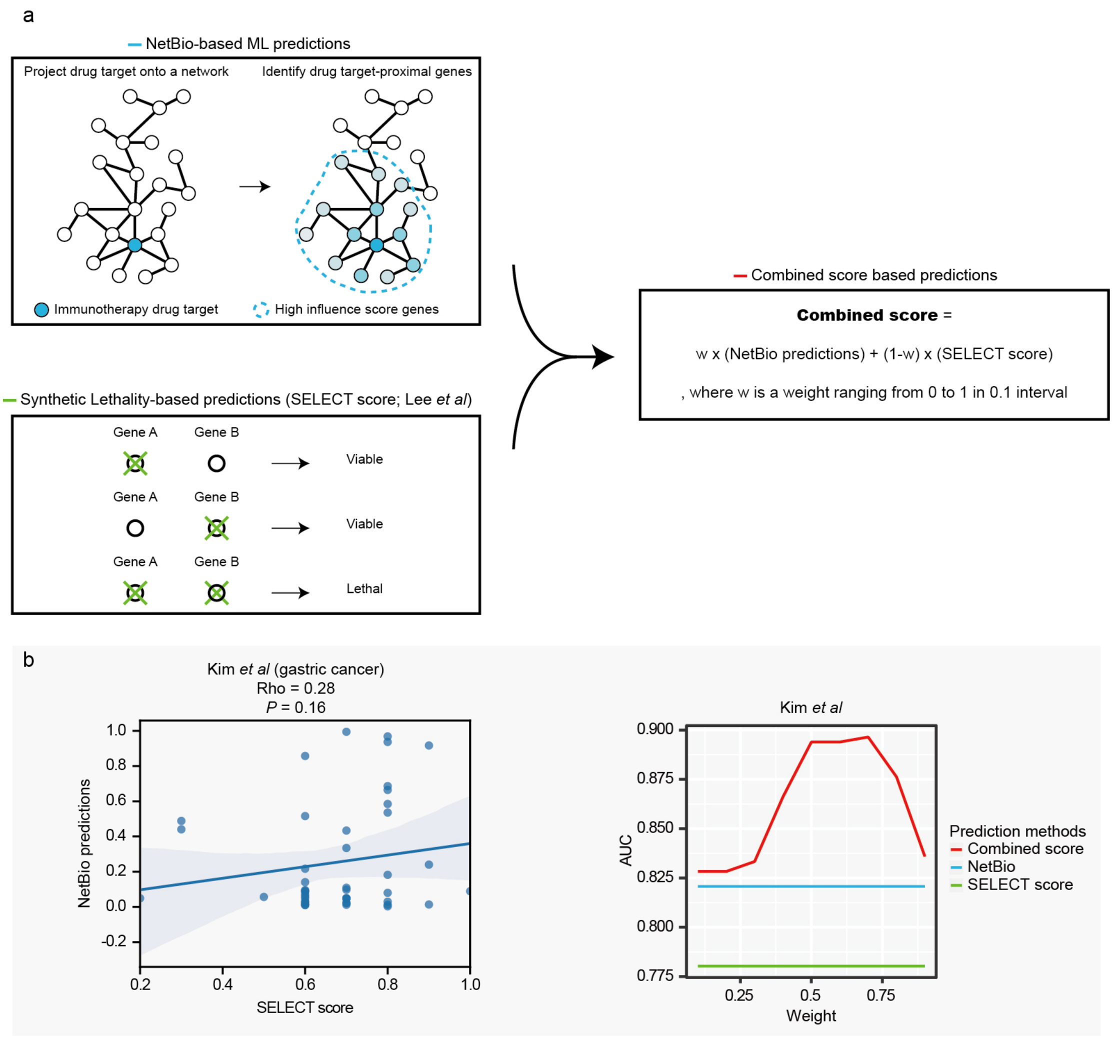


**Supplementary Figure 30. Prediction performance from combining NetBio-based predictions and synthetic lethality-based predictions (SELECT score; Lee *et al.*). (a)** Overall scheme of combining two distinct prediction scores. (**b**) (Left) Correlation between NetBio predictions and the SELECT score and (Right) prediction performance of NetBio, the SELECT score, and combined model for the Kim *et al.* (gastric cancer) cohort. The two-sided Spearman's correlation was used to calculate rho and statistical significance. The lightblue area indicated 95% confidence interval of linear regression line.

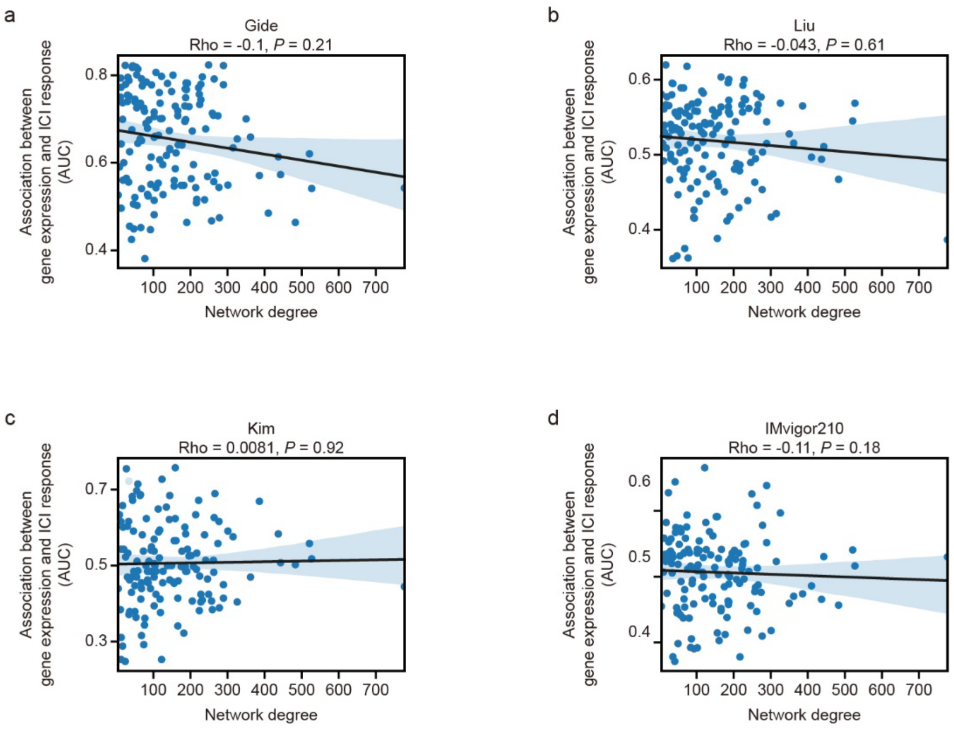


**Supplementary Figure 31. Correlation between network degree of ICI drug targets (PD1, PD-L1, and CTLA4) and ICI efficacy.** (**a**)–(**d**) Correlation between drug efficacy, determined by area under the receiver operating characteristic curve (AUC) based on gene expression levels and ICI response, and degree centrality. Correlation was computed using Spearman's rank correlation. Linear regression line is shown in black. The light-colored area indicated 95% confidence interval of the linear regression line.

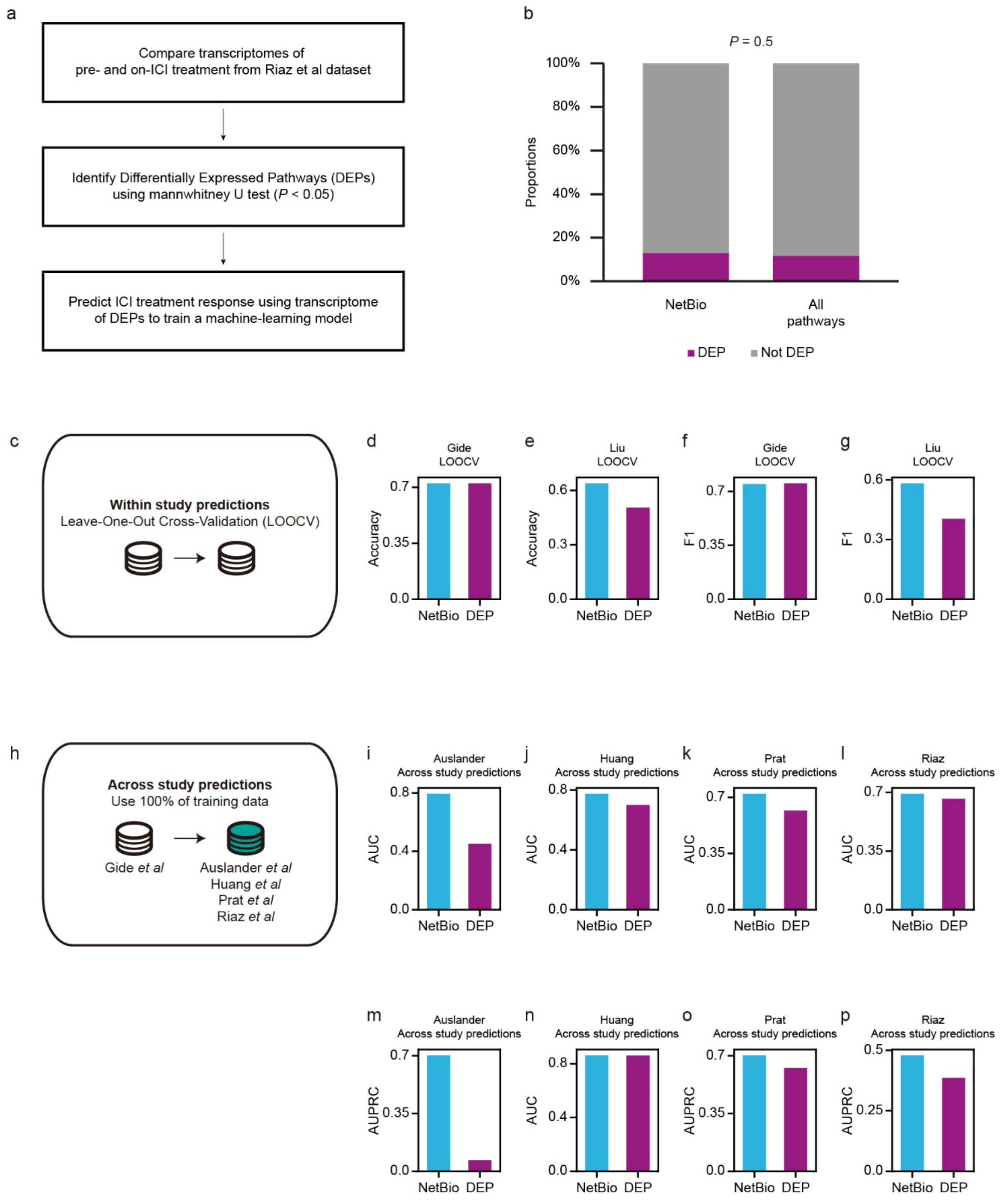


**Supplementary Figure 32. Identification of differentially expressed pathways (DEPs) prior to and during ICI treatment, and predictive performance based on expression levels of DEPs**. (**a**) Overall scheme for identifying DEPs and conducting DEP-based ICI response

prediction. Mann-Whitney U test was used to identify DEPs ($P<0.05$ considered significant). (**b**) Enrichment of DEPs in NetBio pathways. The two-sided Fisher's exact test was used to measure statistical significance. (**c**)–(**g**) Predictive performance of within-study cross validation in the Gide and Liu datasets using (d)–(e) accuracy and (f)–(g) F1 score to quantify predictive performance. (**h**)–(**p**) Across-study predictive performance. The Gide dataset was used to train a machine-learning model, and the Auslander, Huang, Prat, or Riaz datasets were used to test the predictive performance. To quantify performance, we used (i)–(l) area under the receiver operating characteristic curve (AUC) and (m)–(p) area under the precision-recall curve (AUPRC).

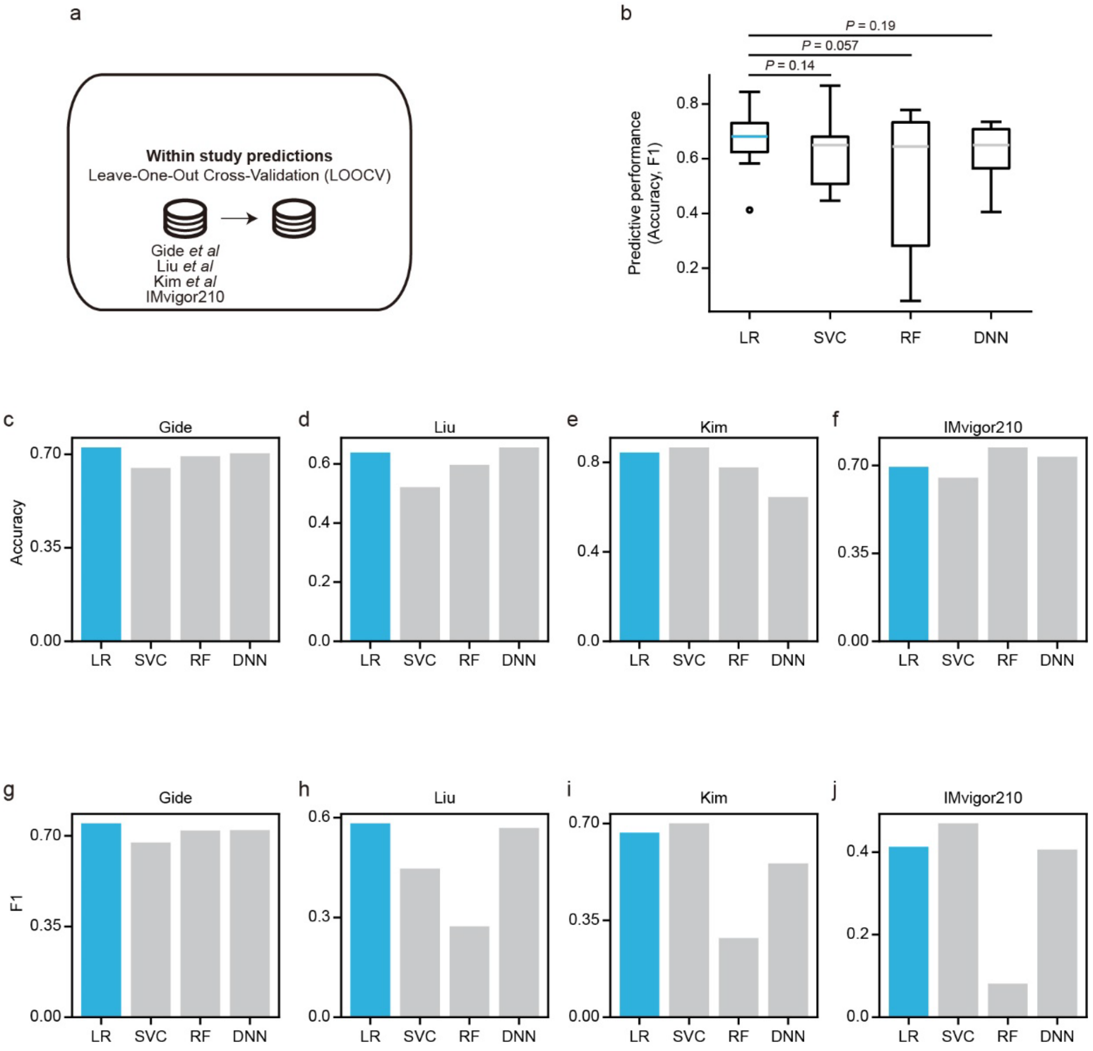


**Supplementary Figure 33. Comparison of LOOCV performance using NetBio machine-learning models based on Logistic Regression (LR), support vector machine classifier (SVC), random forest (RF), or deep neural network (DNN).** (**a**) Overall scheme of immunotherapy response prediction using LOOCV. (**b**) Summarized classification results. Statistical significance was measured using the two-sided paired Student's t-test. Boxplot shows median value, interquartile range (IQR) as bounds of the box and whiskers that extends from the box to upper/lower quartile ± IQR×1.5. (**c**)–(**j**) LOOCV predictive performance

based on (c)–(f) accuracy and (g)–(j) F1 score. 91, 119, 45, 298 independent samples were used for Gide, Liu, Kim and IMvigor210 datasets, respectively.

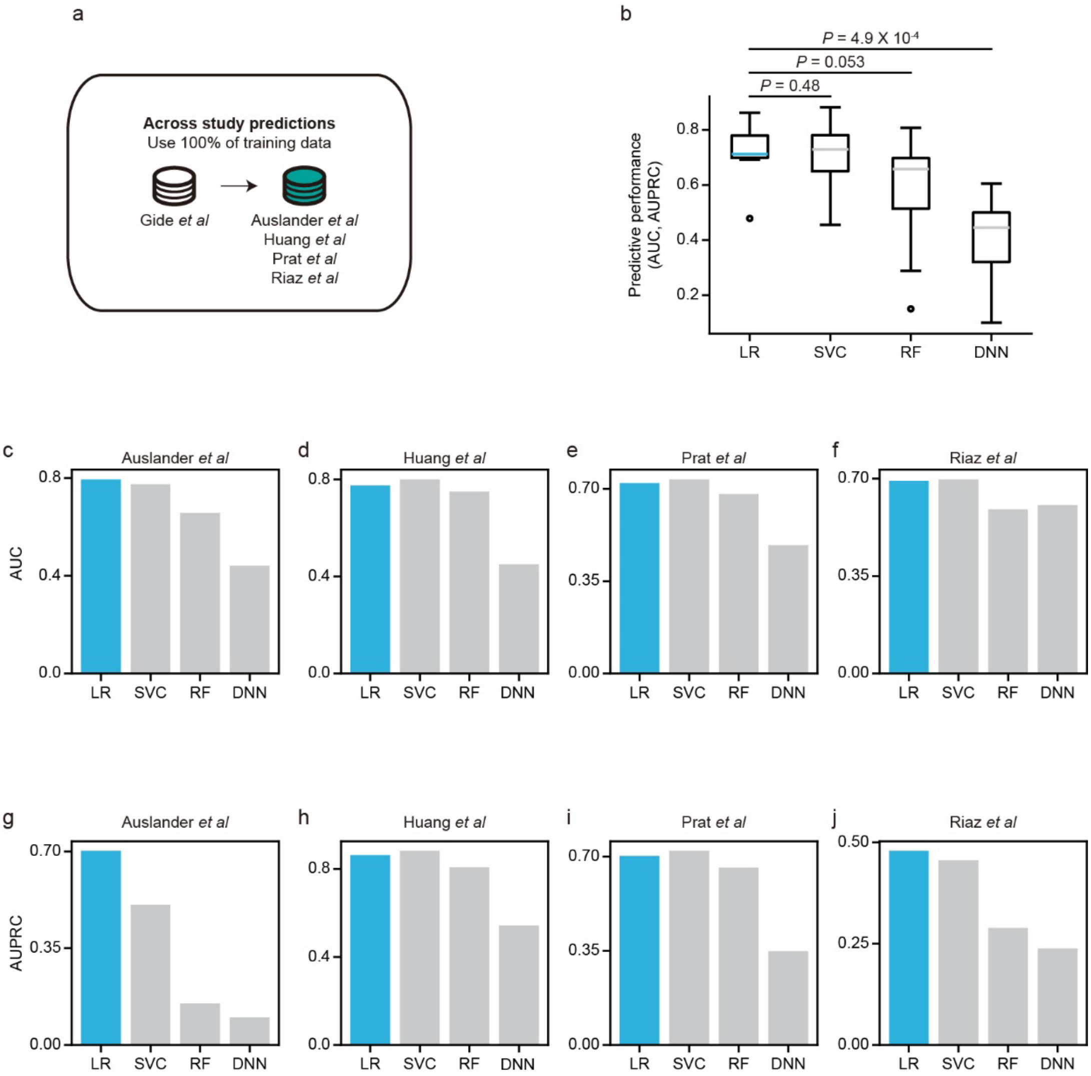


**Supplementary Figure 34. Comparison of across-study predictive performance using NetBio machine-learning models based on Logistic Regression (LR), support vector machine classifier (SVC), random forest (RF), or deep neural network (DNN).** (**a**) Overall scheme of across-study immunotherapy response prediction. (**b**) Summarized classification results. Statistical significance was measured using the two-sided paired Student's t-test. Boxplot shows median value, interquartile range (IQR) as bounds of the box and whiskers that extends from the box to upper/lower quartile ± IQR×1.5. (**c**)–(**j**) Across-study predictive performances using (c)–(f) AUC and (g)–(j) AUPRC. 37, 13, 25 and 49 independent samples

were used for Auslander, Huang, Prat and Riaz datasets, respectively.

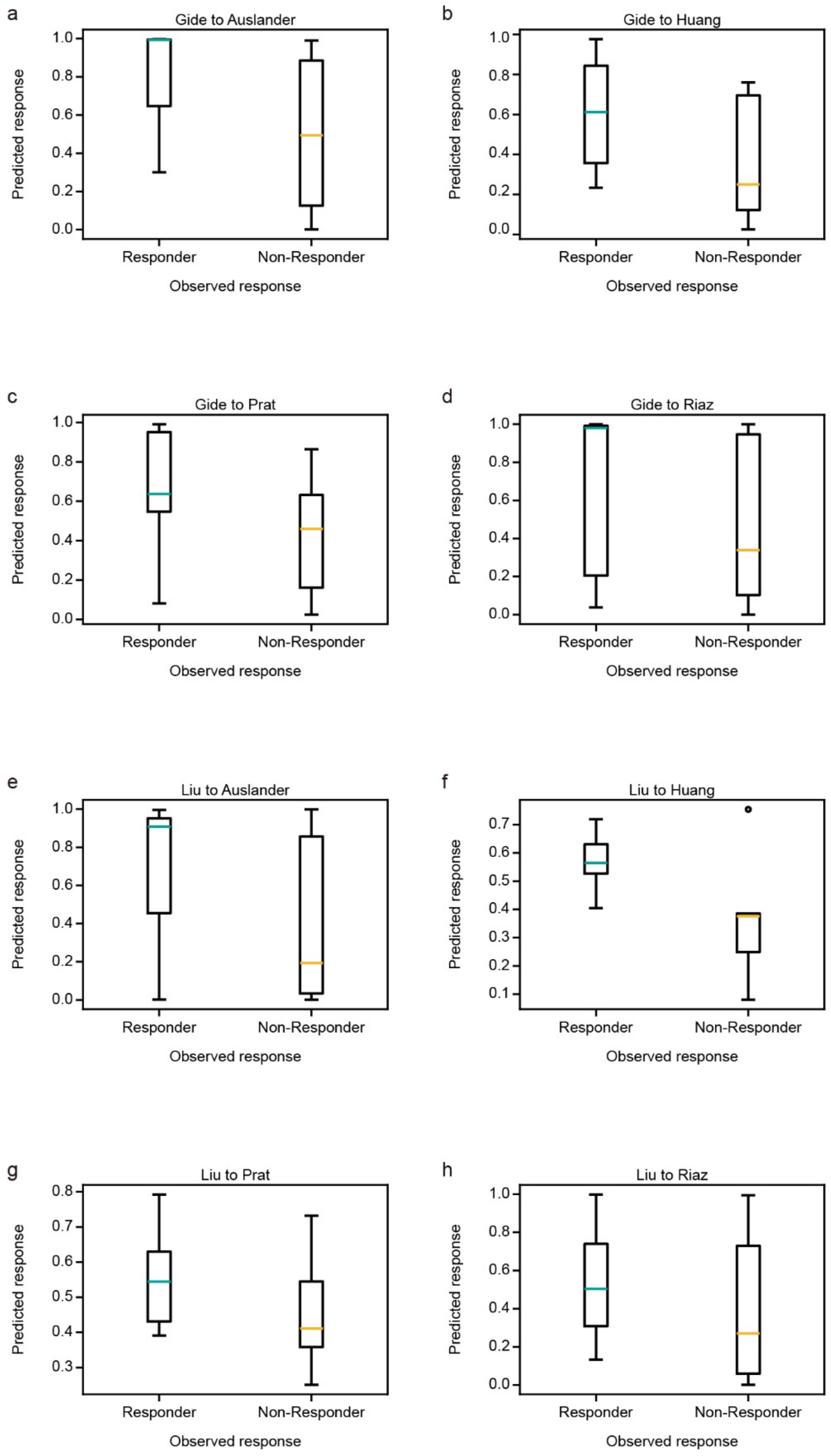


**Supplementary Figure 35. Response probability of across-study predictions using a NetBio-based logistic regression model**. (**a**)–(**h**) Response probability using the (a)–(d) Gide

or (e)–(h) Liu data to train the machine-learning model. 37, 13, 25 and 49 independent samples were used for Auslander, Huang, Prat and Riaz datasets, respectively. Boxplot shows median value, interquartile range (IQR) as bounds of the box and whiskers that extends from the box to upper/lower quartile $\pm$ IQR×1.5.

| Cohort name | Cancer type | Drug | Drug response label | Within or across study analysis |
|---|---|---|---|---|
| Liu | melanoma | Nivolumab, Pembrolizumab | RECIST | within study, across study |
| Gide | melanoma | Pembrolizumab, Nivolumab, Ipilimumab | RECIST | within study, across study |
| IMvigor210 | bladder cancer | Atezolizumab | RECIST | within study |
| Kim | gastric cancer | Pembrolizumab | RECIST | within study |
| Auslander | melanoma | anti-PD-1, anti-CTLA-4 | responder / non-responder | across study |
| Riaz | melanoma | Nivolumab | RECIST | across study |
| Prat | melanoma | Nivolumab, Pembrolizumab | RECIST | across study |
| Huang | melanoma | Pembrolizumab | recurrence | across study |

**Supplementary Table 1. Cohorts and drug response labels used in this study.** For cohorts that used RECIST criteria, we considered patients with Complete Response (CR) or Partial Response (PR) as responders and those with Stable Disease (SD) or Progressive Disease (PD) as non-responders.

| study | markers | accuracy | precision | F1 | TP | TN | FP | FN | sensitivity | specificity |
|---|---|---|---|---|---|---|---|---|---|---|
| Gide | NetBio | **0.73** | 0.74 | **0.75** | 37 | 29 | 13 | 12 | **0.76** | 0.69 |
| Gide | PD1 | 0.67 | 0.70 | 0.69 | 33 | 28 | 14 | 16 | 0.67 | 0.67 |
| Gide | PD-L1 | 0.71 | 0.74 | 0.73 | 35 | 30 | 12 | 14 | 0.71 | 0.71 |
| Gide | CTLA4 | 0.66 | 0.70 | 0.67 | 32 | 28 | 14 | 17 | 0.65 | 0.67 |
| Gide | GeneBio | 0.71 | 0.74 | 0.73 | 35 | 30 | 12 | 14 | 0.71 | 0.71 |
| Gide | CD8T | 0.71 | **0.76** | 0.72 | 34 | 31 | 11 | 15 | 0.69 | **0.74** |
| Gide | T exhaust | 0.68 | 0.70 | 0.71 | 35 | 27 | 15 | 14 | 0.71 | 0.64 |
| Gide | CAF | 0.52 | 0.55 | 0.55 | 27 | 20 | 22 | 22 | 0.55 | 0.48 |
| Gide | TAM | 0.64 | 0.66 | 0.67 | 33 | 25 | 17 | 16 | 0.67 | 0.60 |
| Gide | TME-Bio | 0.65 | 0.67 | 0.68 | 34 | 25 | 17 | 15 | 0.69 | 0.60 |

**Supplementary Table 2. Prediction performances of LOOCV using the Gide dataset to train a machine-learning model (l2 regularized logistic regression).**

| study | markers | accuracy | precision | F1 | TP | TN | FP | FN | sensitivity | specificity |
|---|---|---|---|---|---|---|---|---|---|---|
| Liu | NetBio | 0.64 | 0.54 | **0.58** | 30 | 46 | 26 | 17 | **0.64** | 0.64 |
| Liu | PD1 | 0.07 | 0.03 | 0.03 | 2 | 6 | 66 | 45 | 0.04 | 0.08 |
| Liu | PD-L1 | 0.42 | 0.30 | 0.32 | 16 | 34 | 38 | 31 | 0.34 | 0.47 |
| Liu | CTLA4 | 0.55 | 0.45 | 0.50 | 26 | 40 | 32 | 21 | 0.55 | 0.56 |
| Liu | GeneBio | 0.52 | 0.40 | 0.40 | 19 | 43 | 29 | 28 | 0.40 | 0.60 |
| Liu | CD8T | 0.60 | 0.49 | 0.53 | 27 | 44 | 28 | 20 | 0.57 | 0.61 |
| Liu | T exhaust | **0.65** | **0.55** | 0.57 | 28 | 49 | 23 | 19 | 0.60 | **0.68** |
| Liu | CAF | 0.52 | 0.42 | 0.47 | 25 | 37 | 35 | 22 | 0.53 | 0.51 |
| Liu | TAM | 0.54 | 0.42 | 0.43 | 21 | 43 | 29 | 26 | 0.45 | 0.60 |
| Liu | TME-Bio | 0.55 | 0.43 | 0.46 | 23 | 42 | 30 | 24 | 0.49 | 0.58 |

**Supplementary Table 3. Prediction performances of LOOCV using the Liu dataset to train a machine-learning model (l2 regularized logistic regression).**

| study | markers | accuracy | precision | F1 | TP | TN | FP | FN | sensitivity | specificity |
|---|---|---|---|---|---|---|---|---|---|---|
| Kim | NetBio | **0.84** | **0.78** | **0.67** | 7 | 31 | 2 | 5 | 0.58 | **0.94** |
| Kim | PD1 | 0.51 | 0.27 | 0.35 | 6 | 17 | 16 | 6 | 0.50 | 0.52 |
| Kim | PD-L1 | 0.31 | 0.28 | 0.44 | 12 | 2 | 31 | 0 | **1.00** | 0.06 |
| Kim | CTLA4 | 0.44 | 0.31 | 0.47 | 11 | 9 | 24 | 1 | 0.92 | 0.27 |
| Kim | GeneBio | 0.40 | 0.27 | 0.40 | 9 | 9 | 24 | 3 | 0.75 | 0.27 |
| Kim | CD8T | 0.67 | 0.43 | 0.55 | 9 | 21 | 12 | 3 | 0.75 | 0.64 |
| Kim | T exhaust | 0.82 | 0.75 | 0.60 | 6 | 31 | 2 | 6 | 0.50 | 0.94 |
| Kim | CAF | 0.60 | 0.33 | 0.40 | 6 | 21 | 12 | 6 | 0.50 | 0.64 |
| Kim | TAM | 0.73 | 0.50 | 0.50 | 6 | 27 | 6 | 6 | 0.50 | 0.82 |
| Kim | TME-Bio | 0.82 | 0.70 | 0.64 | 7 | 30 | 3 | 5 | 0.58 | 0.91 |

**Supplementary Table 4. Prediction performances of LOOCV using the Kim dataset to train a machine-learning model (l2 regularized logistic regression).**

| study | markers | accuracy | precision | F1 | TP | TN | FP | FN | sensitivity | specificity |
|---|---|---|---|---|---|---|---|---|---|---|
| IMvigor210 | NetBio | **0.69** | **0.37** | **0.41** | 32 | 175 | 55 | 36 | 0.47 | 0.76 |
| IMvigor210 | PD1 | 0.64 | 0.30 | 0.36 | 30 | 160 | 70 | 38 | 0.44 | 0.70 |
| IMvigor210 | PD-L1 | 0.66 | 0.28 | 0.29 | 21 | 176 | 54 | 47 | 0.31 | **0.77** |
| IMvigor210 | CTLA4 | 0.62 | 0.28 | 0.33 | 28 | 157 | 73 | 40 | 0.41 | 0.68 |
| IMvigor210 | GeneBio | 0.58 | 0.21 | 0.25 | 21 | 152 | 78 | 47 | 0.31 | 0.66 |
| IMvigor210 | CD8T | 0.65 | 0.30 | 0.34 | 27 | 166 | 64 | 41 | 0.40 | 0.72 |
| IMvigor210 | T exhaust | 0.57 | 0.25 | 0.31 | 29 | 141 | 89 | 39 | 0.43 | 0.61 |
| IMvigor210 | CAF | 0.48 | 0.26 | 0.38 | 47 | 97 | 133 | 21 | **0.69** | 0.42 |
| IMvigor210 | TAM | 0.64 | 0.31 | 0.39 | 34 | 156 | 74 | 34 | 0.50 | 0.68 |
| IMvigor210 | TME-Bio | 0.60 | 0.27 | 0.33 | 29 | 150 | 80 | 39 | 0.43 | 0.65 |

**Supplementary Table 5. Prediction performances of LOOCV using the IMvigor210 dataset to train a machine-learning model (l2 regularized logistic regression).**

| Hyperparameter type | Grid |
|---|---|
| *Hidden layers* | 1 x 512, 2 x 256, 3 x 128 |
| *Input dropout ratio* | 0, 0.1, 0.5 |
| *Hidden dropout ratio* | 0.1, 0.3, 0.5 |
| *L2 penalty* | 1e-2, 1e-4 |
| *Epochs* | 10, 50, 100, 200 |

**Supplementary Table 6. The hyperparameter grid used for a deep neural network-based machine-learning model.**
