# Supplementary material for: Network-based machine learning approach to predict immunotherapy response in cancer patients
Source: Nat Commun. 2022 Jun 28;13:3703. doi: 10.1038/s41467-022-31535-6 (PMC9240063; doi:10.1038/s41467-022-31535-6)
Supplement: Supplementary file 4 — Reporting Summary [file 41467_2022_31535_MOESM4_ESM.pdf]

nature portfolio

Corresponding author(s): Sanguk Kim

Last updated by author(s): Jun 16, 2022

Reporting Summary

Nature Portfolio wishes to improve the reproducibility of the work that we publish. This form provides structure for consistency and transparency in reporting. For further information on Nature Portfolio policies, see our Editorial Policies and the Editorial Policy Checklist.

Statistics

For all statistical analyses, confirm that the following items are present in the figure legend, table legend, main text, or Methods section.

n/a	Confirmed	
☐	☒	The exact sample size (*n*) for each experimental group/condition, given as a discrete number and unit of measurement
☐	☒	A statement on whether measurements were taken from distinct samples or whether the same sample was measured repeatedly
☐	☒	The statistical test(s) used AND whether they are one- or two-sided<br>*Only common tests should be described solely by name; describe more complex techniques in the Methods section.*
☐	☒	A description of all covariates tested
☐	☒	A description of any assumptions or corrections, such as tests of normality and adjustment for multiple comparisons
☐	☒	A full description of the statistical parameters including central tendency (e.g. means) or other basic estimates (e.g. regression coefficient) AND variation (e.g. standard deviation) or associated estimates of uncertainty (e.g. confidence intervals)
☐	☒	For null hypothesis testing, the test statistic (e.g. *F*, *t*, *r*) with confidence intervals, effect sizes, degrees of freedom and *P* value noted<br>*Give P values as exact values whenever suitable.*
☒	☐	For Bayesian analysis, information on the choice of priors and Markov chain Monte Carlo settings
☒	☐	For hierarchical and complex designs, identification of the appropriate level for tests and full reporting of outcomes
☐	☒	Estimates of effect sizes (e.g. Cohen's *d*, Pearson's *r*), indicating how they were calculated

Our web collection on statistics for biologists contains articles on many of the points above.

Software and code

Policy information about availability of computer code

Data collection	We used TCGAbiolinks R package (v 2.16.0) to download TCGA dataset.
Data analysis	All analyses were done in python 3.6.12. Python packages used are pandas (1.1.15), numpy (1.19.2), scipy {1.5.4), matplotlib (3.3.3), sklearn (0.24.2), lifelines (0.25.7), networkx (2.5), statsmodels (0.12.2) and pytorch (1.7.l+cu110). PageRank algorithm is included in networkx python module. Source codes are available at a GitHub repository (https://github.com/SBllab/NetBio).

For manuscripts utilizing custom algorithms or software that are central to the research but not yet described in published literature, software must be made available to editors and reviewers. We strongly encourage code deposition in a community repository (e.g. GitHub). See the Nature Portfolio guidelines for submitting code & software for further information.

Data

Policy information about availability of data

All manuscripts must include a data availability statement. This statement should provide the following information, where applicable:

- Accession codes, unique identifiers, or web links for publicly available datasets
- A description of any restrictions on data availability
- For clinical datasets or third party data, please ensure that the statement adheres to our policy

Data Availability statement

For the Gide, Huang, Kim, and Liu datasets, we used normalized expression values and drug responses provided by Lee et al. [https://zenodo.org/record/4661265]. The IMvigor210 dataset was downloaded from the original manuscript [http://research-pub.gene.com/IMvigor210CoreBiologies/]. The Auslander dataset and Riaz dataset were downloaded from the GEO repository under the accession numbers GSE115821 [https://www.ncbi.nlm.nih.gov/geo/query/acc.cgi?acc=GSE115821]

and GSE91061 [https://www.ncbi.nlm.nih.gov/geo/query/acc.cgi?acc=GSE91061], respectively. The Prat dataset was downloaded from the supplementary material of the original manuscript (Table S1). The TCGA datasets were downloaded using the TCGAbiolinks R package [https://bioconductor.org/packages/release/bioc/html/TCGAbiolinks.html]. The human PPI network was downloaded from the STRING database v.11.0. [https://string-db.org/]. The Reactome pathways were downloaded from the MSigDB database. All data used in this study are publically available. Source Data are provided with this paper.

Field-specific reporting

Please select the one below that is the best fit for your research. If you are not sure, read the appropriate sections before making your selection.

☒ Life sciences ☐ Behavioural & social sciences ☐ Ecological, evolutionary & environmental sciences

For a reference copy of the document with all sections, see nature.com/documents/nr-reporting-summary-flat.pdf

Life sciences study design

All studies must disclose on these points even when the disclosure is negative.

Sample size	All of the samples were downloaded from public databases. We used eight different cohorts of immunotherapy-treated cancer patients enough to validate our approach. For leave-one-out cross-validation (within-study analysis), we utilized four cohorts that agree with the following criteria: (i) cohorts with more than 30 samples and (ii) at least 10 samples for both responders and non-responders. (Gide et al., Liu et al., Kim et al., IMvigor210)<br>To validate the ability of our approach to generalize to new datasets (across-study analysis), we tested our approach in four independent datasets. (Auslander et al., Prat et al., Riaz et al., Huang et al.)
Data exclusions	No dataset was excluded.
Replication	We validated our approach in eight different cohorts of immunotherapy-treated cancer patients.
Randomization	Randomization was not relevant to our study since we used only publicly available datasets and didn't generate any new controlled trials.
Blinding	Patients were divided into responders and non-responders based on their response to immunotherapy and thus, no blinding was neccessary. Moreover, we only used publicly available data and therefore, blinding was not relevant to our study.

Reporting for specific materials, systems and methods

We require information from authors about some types of materials, experimental systems and methods used in many studies. Here, indicate whether each material, system or method listed is relevant to your study. If you are not sure if a list item applies to your research, read the appropriate section before selecting a response.

Materials & experimental systems

n/a	Involved in the study	
☒	☐	Antibodies
☒	☐	Eukaryotic cell lines
☒	☐	Palaeontology and archaeology
☒	☐	Animals and other organisms
☐	☒	Human research participants
☒	☐	Clinical data
☒	☐	Dual use research of concern

Methods

n/a	Involved in the study	
☒	☐	ChIP-seq
☒	☐	Flow cytometry
☒	☐	MRI-based neuroimaging

Human research participants

Policy information about studies involving human research participants

Population characteristics	Sequencing of human participants with melanoma, gastric and bladder cancer was performed by TCGA consortium (PMID: 20393554).
Recruitment	Informed consent was obtained from all human participants.
Ethics oversight	Sequencing of human participants was performed by TCGA consortium under a series of locally approved Institutional Review Board (IRB) protocols as described by the consortium (PMID: 20393554).

Note that full information on the approval of the study protocol must also be provided in the manuscript.
